# Diagnostic Accuracy of AI in Prediction and Assessment of Compromised Free Flaps: Systematic Review and Meta-Analysis

**DOI:** 10.2196/91174

**Published:** 2026-09-22

**Authors:** Kuan-Chen Huang, Melanie J Wang, Yu-Ying Chu, Ren-Wen Huang, Yun-Jui Lu, Cheng-Hung Lin, Chung-Chen Hsu, Shih-Heng Chen, Yu-Te Lin, Che-Hsiung Lee

**Affiliations:** 1 Department of Surgery, Chang Gung Memorial Hospital, Taoyuan, Taiwan; 2 Department of Surgery, Section of Plastic Surgery, University of Michigan, Ann Arbor, MI, United States; 3 Department of Plastic and Reconstructive Surgery, Chang Gung Memorial Hospital, No. 5, Fuxing Street, Linkou District, Taoyuan City 333, Taiwan (R.O.C.), Taoyuan, Taiwan, 886 3-318-4301; 4 International Master Science Program in Reconstructive Microsurgery, Chang Gung University, College of Medicine, Taoyuan, Taiwan; 5 Center for Vascularized Composite Allotransplantation, Chang Gung Memorial Hospital, Taoyuan, Taiwan

**Keywords:** free flap assessment, artificial intelligence, diagnostic accuracy, systematic review, meta-analysis

## Abstract

**Background:**

Vascular compromise remains the leading cause of free-flap failure. AI-based monitoring and prediction tools have emerged as a promising adjunct for postoperative free flap monitoring and early detection of vascular compromise. Previous systematic reviews included limited evidence or broadly evaluated reconstructive outcomes.

**Objective:**

This systematic review and meta-analysis assessed the diagnostic accuracy of AI for postoperative free flap monitoring and flap compromise detection.

**Methods:**

Following PRISMA-DTA (Preferred Reporting Items for Systematic Reviews and Meta-Analyses of Diagnostic Test Accuracy Studies), PubMed, Embase, Cochrane, Web of Science, and Scopus were searched from inception to June 21, 2026. Studies developing or validating AI models for free flap monitoring or compromise prediction were included. Pooled sensitivity, specificity, area under the curve (AUC), and diagnostic odds ratio (DOR) were estimated using a hierarchical bivariate random-effects model. Risk-of-bias and applicability were assessed with Quality Assessment of Diagnostic Accuracy Studies-2 (QUADAS-2), and the certainty of evidence was evaluated using the GRADE (Grading of Recommendations Assessment, Development and Evaluation) approach. Prespecified subgroup analyses by modality, model-fit diagnostics, sensitivity analyses, and publication-bias testing with Deeks funnel plot were undertaken. The protocol was prospectively registered in PROSPERO (CRD420251175572).

**Results:**

Of 2098 records identified, 18 studies met the inclusion criteria. Of these, 17 studies were included in the quantitative synthesis. Pooled analysis demonstrated an overall sensitivity of 0.83 (95% CI 0.70‐0.91; prediction interval [PI] 0.21‐0.99), specificity of 0.87 (95% CI 0.65‐0.96; PI 0.03‐1.00), AUC of 0.92 (95% CI 0.90‐0.94), and DOR 36.17 (95% CI 8.27‐158.26; PI 0.09‐14886.43). Image-based AI models demonstrated superior performance, with a sensitivity of 0.92 (95% CI 0.81‐0.97; PI 0.43‐0.99), specificity of 0.95 (95% CI 0.86‐0.98; PI 0.43‐1.00), and AUC of 0.98 (95% CI 0.96‐0.99). Nonimage-based models had sensitivity (0.69, 95% CI 0.45‐0.86; PI 0.11‐0.98) specificity (0.72, 95% CI 0.18‐0.97; PI 0.00‐1.00), and AUC (0.79, 95% CI 0.75‐0.82). For vascular compromise detection, pooled sensitivity was 0.92 (95% CI 0.81‐0.97; PI 0.44‐0.99) and specificity was 0.91 (95% CI 0.79‐0.97; PI 0.35‐1.00). Between-study heterogeneity was substantial, QUADAS-2 identified methodological concerns in several studies, and GRADE rated the certainty of evidence as low.

**Conclusions:**

AI demonstrated favorable diagnostic performance for detecting free flap vascular compromise, particularly with image-based models. These findings support the use of AI as an adjunct to conventional postoperative flap monitoring. Nevertheless, the low certainty of evidence, substantial heterogeneity, and limited external validation indicate that prospective multicenter validation and standardized reporting are required before routine clinical implementation.

## Introduction

Microsurgical free tissue transfer has become a cornerstone of modern reconstructive surgery and is routinely performed in breast, head and neck, extremity, and other complex reconstructions [1,2]. With reported success rates of 94%‐99%, it offers durable and functional restoration for even the most challenging defects [1-3]. Despite continued technical refinements and attentive perioperative care, complications remain a persistent concern [4]. Among these, flap failure is particularly devastating. It is most often caused by vascular compromise, including arterial insufficiency, venous congestion, and ischemia-reperfusion injury, with incidence rates reported in 10%‐30% of cases [5]. These events frequently necessitate reoperation, with 2%‐5% of flaps requiring surgical exploration, thereby increasing morbidity, prolonging hospitalization, escalating healthcare costs, and imposing substantial psychological stress on patients [6-8].

Because salvage success is highly dependent on the interval between vascular compromise and reintervention, accurate risk identification, early recognition of compromised flaps, and timely response are critical [9]. Risk stratification and vigilant postoperative monitoring remain essential components of high-quality microsurgical care [10]. However, current assessment relies heavily on clinical observation, typically performed by staff and interpreted by experienced microsurgeons [6,11]. These approaches are labor-intensive, require continuous bedside monitoring, and are inherently subjective, often ambiguous and challenging to interpret. Identifying key predictive factors and prioritizing high-risk patients for intensified monitoring may reduce the risk of flap failure and improve microsurgical outcomes [12-14].

In recent years, advances in computing and data science have accelerated the integration of AI into clinical workflows [15]. AI systems are capable of emulating aspects of human perception and reasoning while reducing fatigue-related errors and certain systematic biases [16]. Among these, convolutional neural networks, which process images through hierarchically arranged filters analogous to the visual cortex, have demonstrated utility in medical imaging tasks. These include anatomical landmark detection, vascular segmentation, and perforator mapping, where high accuracy is essential for surgical planning [17,18].

Within AI, machine learning, a data-driven approach that enables pattern recognition and predictive modeling, has shown promise in various domains of plastic surgery, including wound analysis, surgical planning, dermatologic diagnosis, and postoperative outcome prediction [17,19-21]. A growing number of studies have also investigated the use of AI in predicting and assessing flap-related complications [22]. While these tools hold considerable promise in augmenting clinical judgment and reducing human error, the diagnostic accuracy and clinical reliability in free flap monitoring remain insufficiently established [23,24]. This uncertainty poses a significant barrier to widespread clinical implementation.

Two systematic reviews [23,24] have recently summarized the application of AI in free flap surgery. However, the available evidence remains incomplete in several respects. First, both reviews framed the outcome broadly as the prediction of postoperative complications rather than the diagnostic performance of AI for real-time flap monitoring and early recognition of vascular compromise, the specific scenario in which the interval to reexploration determines salvage [9]. Second, neither review distinguished image-based surveillance models from clinical variable-based risk-prediction models, although these approaches differ fundamentally in their inputs, intended point of care, and expected diagnostic behavior, and may therefore warrant separate synthesis [22]. Third, both searches predate a rapidly expanding body of image-based monitoring evidence, including camera-integrated deep-learning platforms, optical detection of venous congestion, and remote real-time surveillance systems [25-27]. These gaps underscore the need for an updated diagnostic test accuracy synthesis that reflects current clinical applications of AI and separately evaluates image-based and nonimage-based models according to their intended roles in postoperative free flap care.

Therefore, we conducted a systematic review and meta-analysis to evaluate the diagnostic performance of AI-based approaches in predicting and detecting free flap compromise following microsurgical reconstruction. We also compared the diagnostic performance of image-based and nonimage-based AI models to better characterize their role in postoperative free flap monitoring.

## Methods

### Overview

This systematic review and meta-analysis was registered with PROSPERO (International Prospective Register of Systematic Reviews; CRD420251175572) and conducted in accordance with the PRISMA-DTA (Preferred Reporting Items for Systematic Reviews and Meta-Analyses of Diagnostic Test Accuracy Studies) guidelines [28] and the PRISMA (Preferred Reporting Items for Systematic Reviews and Meta-Analyses) 2020 statement [29]. The study followed Transparency in the reporting of AI (TITAN) recommendations [30]. The literature search was reported in accordance with the PRISMA-S (Preferred Reporting Items for Systematic Reviews and Meta-Analyses literature search extension) extension to enhance the transparency and reproducibility of the search process. The complete search strategies for all databases and the completed PRISMA-S checklist are provided in Multimedia Appendix 1 and Checklist 1, respectively [31].

### Search Strategies

**Figure 1.**
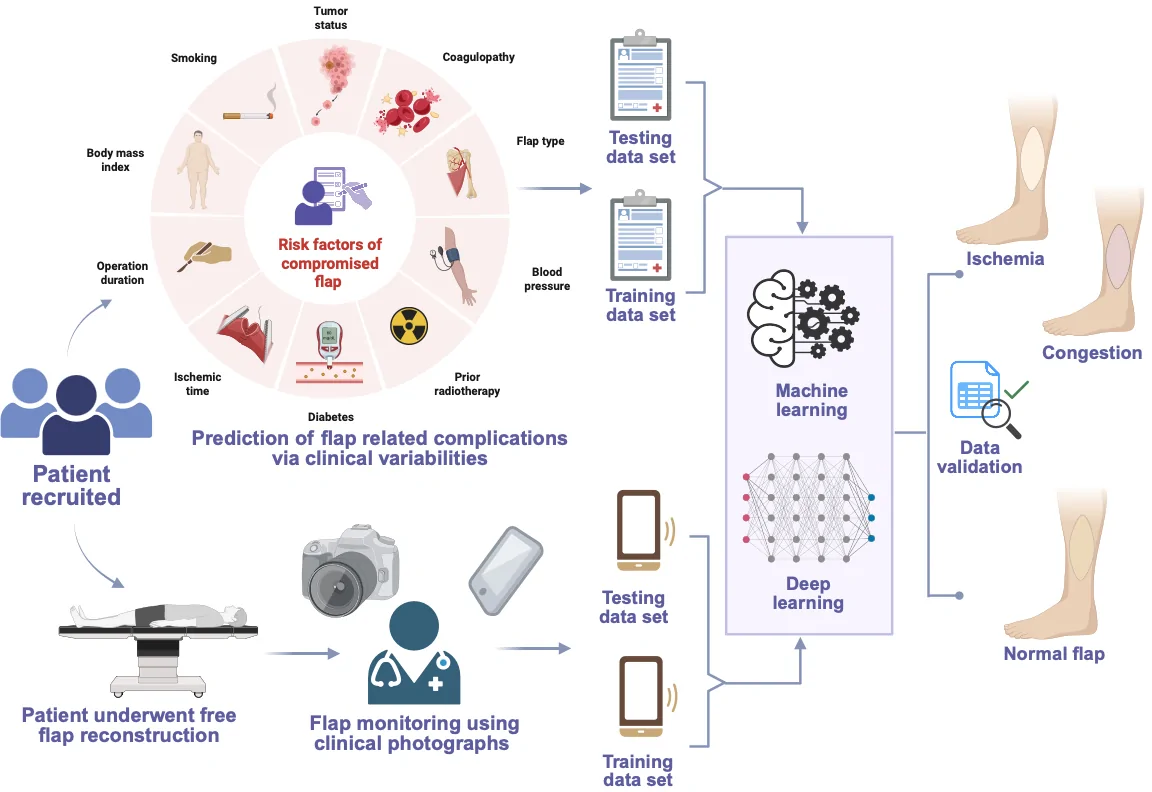
Illustrative diagram [32] of the 2 principal AI–assisted approaches identified in the included studies of microvascular free flap reconstruction.

A comprehensive search was performed across PubMed, Embase, Cochrane, Web of Science, and Scopus databases for studies published up to June 21, 2026, without language restrictions (Figure 1). Database searches were updated before manuscript revision to capture recently published studies. The search was designed to identify studies that developed and validated deep learning or machine learning algorithms for the diagnosis of free flap status following microsurgery, using either imaging or clinical variables. Keywords and MeSH terms included “Artificial Intelligence,” “Deep learning,” “Machine learning,” “Radiomic,” “Automated diagnosis,” “Free flap reconstruction,” “Flap monitoring,” “Flap viability,” “ischemic complications,” and “Flap failure” (Multimedia Appendix 1). Search strategies were developed independently by the investigators and were not adapted from previous systematic reviews [23,24]. Backward citation searching was conducted by manually screening the reference lists of all included studies and relevant review papers. Forward citation searching was also performed to identify studies citing the included papers. No other supplementary search methods were used.

### Selection Criteria

Eligibility assessment was conducted independently by two reviewers (KCH, CHL) who screened titles and abstracts of the search results, with discrepancies resolved through discussions. The inclusion criteria were: (1) original research paper evaluating the performance of AI in identifying or predicting flap compromise, regardless of model type; (2) cohort studies incorporating diagnostic analysis; (3) study populations consisting of patients who underwent microsurgical reconstruction; and (4) clearly defined diagnostic criteria for flap compromise as stated in the study with available and extractable data. Studies were excluded based on the following criteria: (1) nonoriginal study types, including systematic reviews, review papers, case reports, commentaries, and letters; (2) irrelevant research focus, such as basic science experiments; (3) studies lacking AI applications or relevance to flap-related outcomes; (4) incomplete diagnostic data, particularly when key metrics such as true positive (TP), true negative (TN), false positive (FP), and false negative (FN) could not be determined; (5) unavailable full-text articles; and (6) duplicate publications. There were no language restrictions applied in the search strategy.

### Data Extraction and Management

The data extraction framework was developed with reference to the Cochrane Handbook guidance [33,34]. Data extraction was performed independently by two reviewers (KCH and YYC) using a predefined data extraction sheet. Extracted variables included study design, country, year of publication, authorship, patient population characteristics such as gender and age, and model-related characteristics including training and testing sample sizes, validation methods, reference standards, AI model type, and modality of flap assessment. Diagnostic performance metrics, including sensitivity, specificity, and the corresponding TP, FP, TN, and FN counts, were collected. When studies included both animal and human experiments, only human data were extracted for quantitative synthesis. Information regarding missing data handling and imputation methods was also extracted when reported.

Study independence was confirmed based on author group, region, study period, and institution, with no evidence of overlapping cohorts, and analyses were conducted at the patient level with image- or pixel-level data aggregated accordingly to avoid distortion of diagnostic estimates [35]. When multiple models were reported within a study, the best-performing model identified by the original authors was selected. If no preferred model was specified, the algorithm with the highest area under the curve (AUC) was chosen, with priority given to higher sensitivity because of the clinical importance of early flap compromise detection. To avoid overrepresentation of individual cohorts, only one model from each study was included in the pooled analysis. Contingency tables were reconstructed directly from reported results or supplementary data or back calculated from available performance metrics. When necessary, corresponding authors were contacted for clarification or provision of additional data.

### Risk-of-Bias and Quality Assessment

Methodological quality was assessed using the Quality Assessment of Diagnostic Accuracy Studies 2 (QUADAS-2) tool, which evaluates four domains: patient selection, index test, reference standard, and flow and timing. Each domain was rated independently by two reviewers (KCH, MJW) for risk of bias as low, high, or unclear, and the first 3 domains were also appraised for applicability concerns [36]. Discrepancies were adjudicated through discussion or consultation with a third senior reviewer (CHL). The certainty of evidence was assessed using the GRADE (Grading of Recommendations Assessment, Development and Evaluation) approach for diagnostic test accuracy studies [37].

### Diagnostic Performance and Statistical Analysis

Statistical analyses were performed using R (R Foundation for Statistical Computing) and Stata version 18.0 (StataCorp LLC) [38-40]. A bivariate random-effects model was applied to jointly synthesize sensitivity and specificity, generating pooled estimates with corresponding 95% CIs. Diagnostic odds ratios (DOR) were also computed. Overall diagnostic performance was visualized using summary receiver operating characteristic (SROC) curve, with the AUC reported as an indicator of discriminatory capacity, where values approaching 1.0 reflected superior accuracy [41]. For univariate random-effects analyses, the Sidik-Jonkman estimator was used to estimate between-study variance, and the Hartung-Knapp adjustment was applied to calculate the 95% CIs in R. Corresponding 95% prediction intervals (PIs) were calculated and displayed in the forest plots. Sensitivity and specificity were pooled as logit-transformed proportions, whereas DOR and likelihood ratios were pooled on the natural logarithmic scale. For studies containing at least one zero cell among the TP, FP, TN, or FN counts, a continuity correction of 0.5 was added to all four cells before calculation of the DOR, likelihood ratios, and their variances. No continuity correction was applied to studies without zero cells. These supplementary analyses did not replace the primary bivariate diagnostic meta-analysis [42]. Statistical significance was defined as *P*<.05.

### Clinical Value Verification

The clinical utility of AI-based diagnostic approaches was further explored through Fagan plot analysis, which translated pooled diagnostic metrics into posttest probabilities, thereby enabling estimation of their clinical applicability in predicting flap compromise and related complications [43,44]. The pooled prevalence of flap compromise was estimated by dividing the total number of TP and FN cases by the overall sample size (TP+FN+FP+TN) across included studies. Publication bias was assessed using Deeks funnel plot asymmetry test, with significant asymmetry (*P*<.10) interpreted as suggestive of small-study effects or selective reporting.

### Subgroup Analysis

To address potential sources of heterogeneity, prespecified subgroup analyses were conducted according to study methodology and diagnostic focus (Figure 1, illustrating approaches from: first approach used perioperative clinical variables for risk prediction of flap-related outcomes, second approach applied postoperative image-based analysis to identify flap compromise through changes in flap color and surface characteristics). Studies were categorized as image-based models for real-time postoperative flap surveillance and early detection of flap compromise, or nonimage-based models using clinical and perioperative variables for risk stratification and prediction of flap perfusion-related complications. Additional subgroup analyses focused on models specifically designed to detect vascular compromise. Results were presented using SROC curves and forest plots to facilitate subgroup comparisons.

### Heterogeneity and Sensitivity Analysis

Between-study heterogeneity was further quantified using the *I*² statistic, with values greater than 50% considered to represent substantial heterogeneity. PIs were additionally calculated to provide a clinically relevant interpretation of between-study variability [45]. A bivariate box plot was also generated to visually inspect the distribution of sensitivity and specificity and to identify potential outliers.

The robustness of the primary meta-analysis was interrogated through sensitivity analyses, which included assessments of goodness of fit via Q–Q plot of deviance residuals, evaluation of bivariate normality using a Mahalanobis distance-based Q–Q plot, identification of influential studies through Cook distance, and systematic detection of outliers. Outliers were defined as studies with standardized residuals exceeding 2.0 for sensitivity of FP rate, corresponding to a 99% CI (z score±2.0), or those demonstrating significant deviation from expected model fit. A conservative approach was employed to minimize inappropriate exclusions. Identified outliers were removed and the bivariate model was reestimated to determine their impact on pooled performance metrics and heterogeneity. Publication bias was assessed using Deeks funnel plot asymmetry test, with *P*<.10 considered suggestive of significant small-study effects.

### Ethical Considerations

Ethical approval was not required because this study was a systematic review and meta-analysis based exclusively on published data.

## Results

### Literature Search

**Figure 2.**
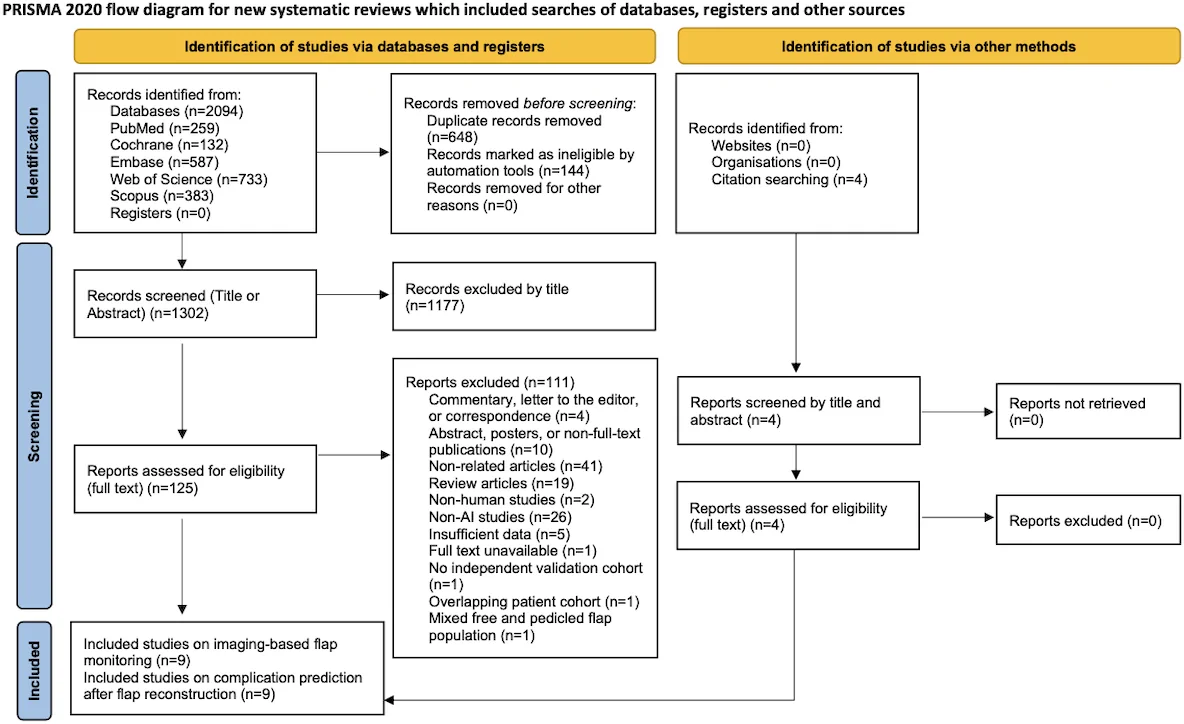
PRISMA (Preferred Reporting Items for Systematic Reviews and Meta-Analyses) flow diagram illustrating study identification, screening, eligibility assessment, and final inclusion for this systematic review and meta-analysis of AI–based models for prediction and surveillance of compromised microvascular free flaps.

The process of study acquisition is illustrated in the PRISMA flow chart (Figure 2). A total of 2098 studies were initially retrieved, of which 792 studies were excluded as duplicates. After screening the remaining 1306 papers by title and abstract, 1177 were excluded, leaving 129 full text studies for detailed assessment. Following eligibility evaluation, 111 studies were excluded, resulting in 18 studies [20,25-27,46-59] ultimately included in this meta-analysis.

Seventeen studies included in the quantitative diagnostic accuracy meta-analysis are summarized in Tables 1 and 2. All evaluated AI for the prediction or detection of free flap compromise. One additional study [59] met the eligibility criteria and is presented in the PRISMA flow diagram and Table 1; however, it was excluded from the quantitative synthesis because insufficient data were available to reconstruct the 2×2 contingency table.

**Table 1.** Baseline characteristics of the included studies evaluating AI–based prediction and surveillance models for compromised microvascular free flaps, including study design, country, patient population, reconstructive indication, flap type, outcome definition, sample size, and validation strategy.

Author, year	Country	Time period	Research type	Included patients (n)	Mean age, range (, years)	Female (%)	Reference standard	Targeted condition	Type of internal validation	External validation
Zhang et al, 2026 [25]	China	NR^a^	Retrospective	7	NR	NR	Clinical evaluation	Vascular complications	Hold-out validation: train–test split validation (4:1 split)	NR
He et al, 2026 [26]	China	January 2018-December 2024	Retrospective	576	57	79.34	Clinical evaluation	Free flap failure	6:2:2 train: validation: test split	NR
Huang et al, 2026 [27]	China	January 2019-June 2025	Retrospective	615	NR	NR	Clinical evaluation	Venous congestion	Patient-level 10-fold cross-validation	Yes
Kim et al, 2026 [58]	South Korea	June 2021-March 2024	Retrospective	131	58.1	38.2	Clinical evaluation	Flap failure	5-fold cross-validation	NR
Geovani et al, 2025 [59]	Indonesia	NR	Preliminary study	NR	NR	NR	Image label: visual assessment	Flap compromise	Train: test split with validation set	NR
Fang et al, 2025 [57]	China	January 2021-January 2024	Retrospective	341	59.41	43.07	Clinical evaluation	Vascular complications	Internal split-sample validation: bootstrap internal validation	NR
Monarchi et al, 2025 [56]	Italy	NR	Retrospective	125	52.3	41.6	Clinical evaluation	Flap takeback	K-fold cross-validation	NR
Maktabi et al, 2025 [55]	Germany	NR	Retrospective	59	NR	NR	Clinical evaluation	Flap malperfusion	Leave-one-patient-out cross-validation (LOPOCV)	Yes
Oleru et al, 2025 [54]	United States	January 2019-January 2024	Retrospective	458	50.5	52.90	Clinical evaluation	Flap takeback due to vascular complications	10-fold cross-validation	NR
Kim et al, 2024 [46]	South Korea	March 2020-August 2023	Prospective	305	62 (8‐86)	41.6	Clinical evaluation	Vascular complications	5-fold cross-validation, Hold-out (train-test split) validation	Yes
Yang et al, 2024 [50]	China	January 2019-December 2021	Retrospective	570	49.5	28.4	Surgical confirmation	Vascular complications	Split-sample (60:40), SMOTE^b^	NR
Wang et al, 2024 [49]	United States	2012‐2019	Retrospective	NR	63 (55, 71)	32	Clinical evaluation	Reoperation	Stratified k-fold cross-validation	Yes
Huang et al, 2023 [47]	Taiwan	January 2021-July 2021	Prospective	176	NR	NR	Clinical evaluation	Vascular complications	10-fold cross-validation	NR
Hsu et al, 2023 [48]	Taiwan	April 2021-March 2022	Retrospective	642	55.7 (43.2‐68.2)	24	Clinical evaluation, surgical confirmation	Venous congestion	Random split of photograph	Yes
Asaad et al, 2023 [51]	United States	January 2005-December 2018	Retrospective	4000	NR	NR	Clinical evaluation	Total flap loss	10-fold cross-validation	NR
Tighe et al, 2022 [52]	United Kingdom	NR	Retrospective	1593	NR	46.7	Clinical evaluation	Complete flap failure	10-fold cross-validation	NR
Shi et al, 2022 [53]	China	January 2006-December 12, 2020	Retrospective	946	42 (range 13‐65)	58.3	Clinical evaluation	Flap failure	5-fold cross-validation	NR
O’Neill et al, 2020 [20]	Canada	2009/01-2017/01	Retrospective	1012	50.9	NR	Clinical evaluation	Total flap failure	Train:test split with 3 ratios (50:50, 60:40, 70:30)	NR

a NR: not reported

b SMOTE: synthetic minority oversampling technique.

Most included studies were retrospective single-center cohort studies, although several investigations incorporated multicenter or externally validated datasets. Table 1 presents the basic study characteristics, including design, patient population, and clinical context, while Table 2 details model specifications, validation methods, and diagnostic performance metrics. Most studies were retrospective (n=15) in design, with only two prospective investigations conducted by Huang et al [47] and Kim et al [46]. External validation was reported in five studies [27,46,48,49,55]. Eight studies [25-27,46-48,55,58] applied image-based analysis, using photographic data to evaluate flap viability primarily based on colorimetric features. The remaining 9 studies [20,49-54,56,57] developed predictive models using diverse clinical variables to identify patients at risk for flap compromise. These models integrated preoperative (eg, laboratory values, neoadjuvant therapy, and medication history), intraoperative details (eg, operative duration, ischemic time, flap selection, and laterality), and postoperative parameters (eg, hypotensive episodes) details. Demographic and clinical factors, including age, BMI, comorbidities, and primary tumor characteristics such as metastatic status, were also incorporated. Although the included studies encompassed a variety of free flap types, the anterolateral thigh (ALT) flap for head and neck reconstruction was the most frequently investigated.

**Table 2.** Detailed overview of AI^a^.

	Algorithm		Data set						Performance					
Author, year	All model use	Best model output	Input	Total data sets	Negative data set	Positive data set	Train/Test data set	Proportion of data set (train: test)	TP^b^	FP^c^	FN^d^	TN^e^	SP^f^ (%)	SE^g^ (%)
Image-based methods														
Zhang et al, 2026 [25]	Multilayer Perceptron, SVM^h^, KMEANS^i^, LR^j^, DT^k^, RF^l^, K-Neighbors, GaussianNB	RF	BVP^m^ signals	76	60	16	NR^n^	4:1	3	0	2	9	100^o^	60^o^
He et al, 2026 [26]	RF, LR, KNN^p^, LDA^q^, DT^r^, AdaBoost, multilayer perceptron, and XGBoost^s^; ResNet, GoogleNet, and DenseNet	ResNet	CIELAB^t^, RGB^u^, HSV^v^ color space	2575	2010	565	NR	6:2	392	12	11	67	84.8	97.2
Huang et al, 2026 [27]	FS-Net^w^ (segmentation), TensorFlow Lite CNN^x^, DLscope^y^	TensorFlow Lite (FS-Net)	RGB images	1649	762	83	342/845	1:2.5^o^	79	20	4	742	97.4^o^	95.2
Kim, 2026[58]	ViT, Siamese network, Dynamic Margin Triplet Loss, Cross-entropy loss	Siamese ViT^z^	Images	1862	368	5	1489/373	4:1	4	2	1	366	99.5^o^	80.0^o^
Maktabi et al, 2025 [55]	RF, MP, SVM LR, CNN (3D data)	CNN	Hyperspectral images	59	48	11	54/4	13:1	8^o^	12^o^	3^o^	36^o^	76	70
Kim et al, 2024 [46]	VGG16^aa^, Custom CNN, ResNet50, InceptionV3, DenseNet121	DenseNet121	Images	11,112	10,115	997	5606/5506	1:1^o^	455	457	45	4549	91.0^o^	91.0^o^
Huang et al, 2023 [47]	KNN, DT, RF, AdaBoost	RF	Images	805	555	250	644/161	8:2	49^o^	1^o^	1^o^	110^o^	99^o^	98^o^
Hsu et al, 2023 [48]	Core ML^ab^	Core ML	Images	1761	1525	236	328/921	1:3^o^	80	39	4	798	95.3^o^	95.2^o^
Clinical variable-based methods														
Fang et al, 2025 [57]	LR, Random forest	LR	Clinical variables	341	259	82	239/102	7:3	16^o^	27^o^	6^o^	53^o^	66.7	73.6
Monarchi et al, 2025 [56]	Unspecified machine learning algorithms	LR	“Modified” HALP^ac^ score	125	115	10	NR	NR	9^o^	9^o^	1^o^	106^o^	92.4	90.9
Oleru et al, 2025 [54]	RF	RF	Clinical variables	458	417	27	NR	6:4	12^o^	36^o^	4^o^	128^o^	78	75
Yang et al, 2024 [50]	LRR, RF, ANN^ad^	ANN	Clinical variables	570	524	46	317/228	6:4	18	47	3	160	77.2^o^	85.7^o^
Wang et al, 2024 [49]	eXtreme Gradient Boosting (XGBoost)	XGBoost	Clinical variables	4931	4027^o^	904	NR	NR	461^o^	1450^o^	443^o^	2577^o^	64	51
Asaad et al, 2023 [51]	LRR, MARS^ae^, KNN, SVM, DT, ANN, RF,	RF	Clinical variables	4000	3932^o^	68^o^	3201/799	8:2	14^o^	785^o^	0^o^	0^o^	0	100
Tighe et al, 2022 [52]	XGBoost +RUSBoost, DF(RF)^af^+RU, DF (Boost; RF)	DF (Boost (RF))	Clinical variables	1593	1517	76	NR	NR	41	658	34	860	56.6^o^	54.6^o^
Shi et al, 2022 [53]	RF, SVM, gradient boosting.	RF	Clinical variables	946	912	34	473/473	1:1	12^o^	3^o^	6^o^	452^o^	99^o^	69
O’Neill et al, 2020 [20]	SMOTE^ag^, ROSE, random under sampling, random oversampling, tree bagging ensemble method	ROSE^ah^	Clinical variables	1012	1000	12	608/404	6:4	2	53	2	347	86.8	50.0

a ”n” represents the unit of analysis reported in each study (images for image-based models and patients for clinical variable–based models). AI algorithms, predictor variables, or imaging inputs, dataset characteristics, imbalance-handling strategies, model development methods, and diagnostic performance metrics across the included studies. The cumulative total may contain an error.

b TP: true positive.

c FP: false positive.

d FN: false negative.

e TN: true negative.

f SP: specificity.

g SE: sensitivity.

h SVM: support vector machine.

i K-means: k-means clustering.

j LR: logistic regression.

k DT: decision tree.

l RF: random forest.

m BVP: boundary value problem.

n NR: not reported.

o Calculated values.

p KNN: K-nearest neighbor.

q LDA: linear discriminant analysis.

r DT: decision tree.

s XGBoost: extreme gradient boosting.

t CIELAB: Commission Internationale de l'éclairage L*a*b*.

u RGB: red, green, blue.

v HSV: hue, saturation, value.

w FS-Net: flap segmentation network.

x CNN: convolutional neural network.

y DL: deep learning.

z ViT: vision transformer.

aa VGG16: Visual Geometry Group 16.

ab ML: machine learning.

ac HALP: hemoglobin, albumin, lymphocyte, and platelet.

ad ANN: artificial neural network.

ae MARS: multivariate adaptive regression splines.

af DF: deep forest.

ag SMOTE: synthetic minority oversampling technique.

ah ROSE: random over sampling examples.

### Risk-of-Bias and Certainty of Evidence

**Figure 3.**
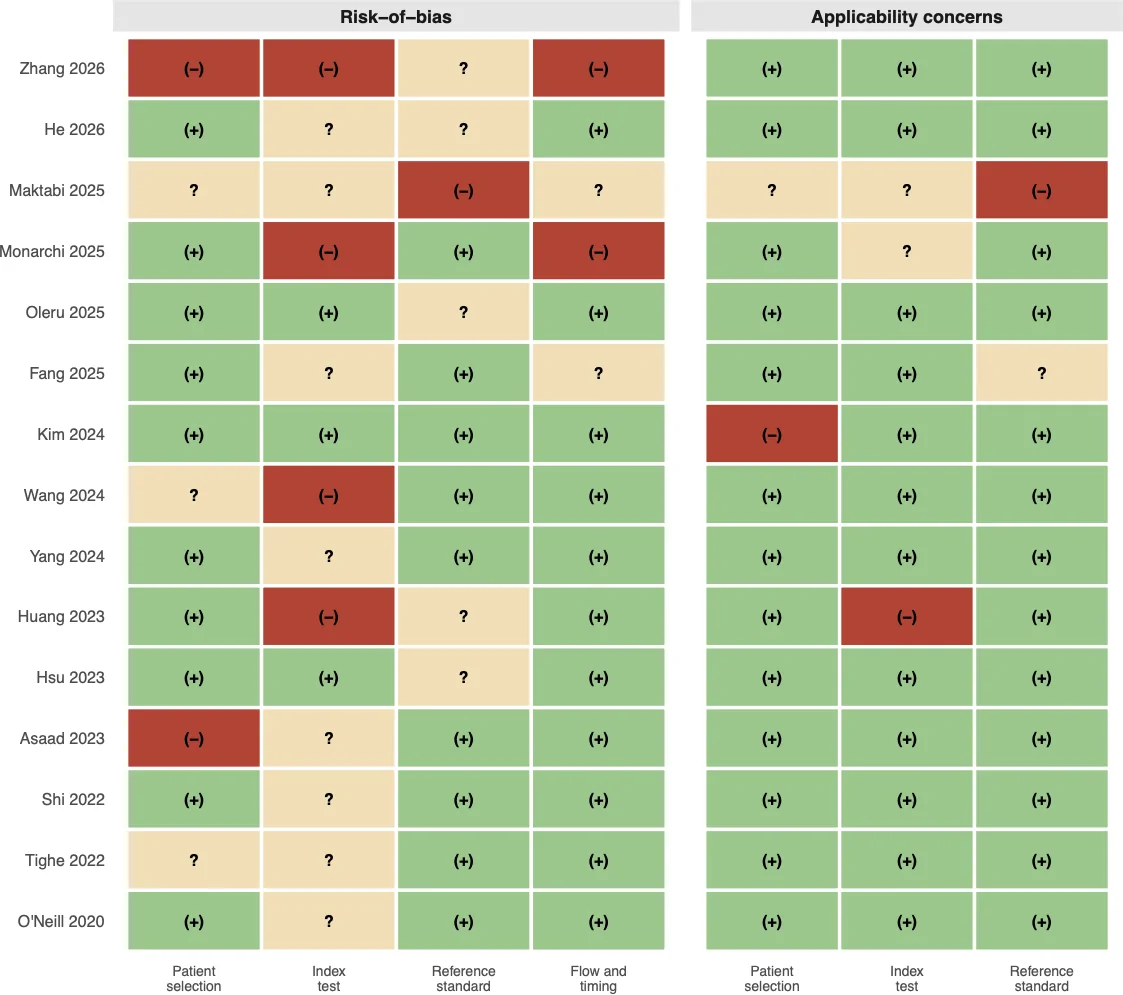
Risk-of-bias and methodological quality assessment using the QUADAS-2 (Quality Assessment of Diagnostic Accuracy Studies-2) tool. Summary chart of risk-of-bias judgments across all domains and studies. Green indicates low risk of bias, yellow indicates unclear risk, and red indicates high risk [20,25-27,46-58].

**Figure 4.**
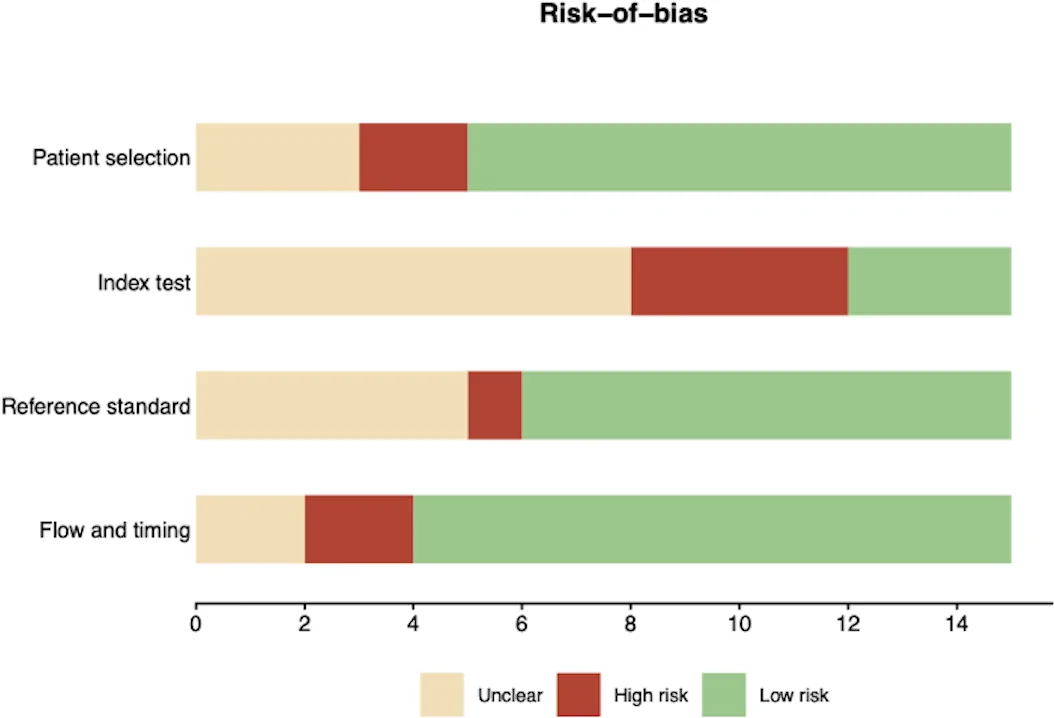
Graphical representation of risk of bias for each individual study.

**Figure 5.**
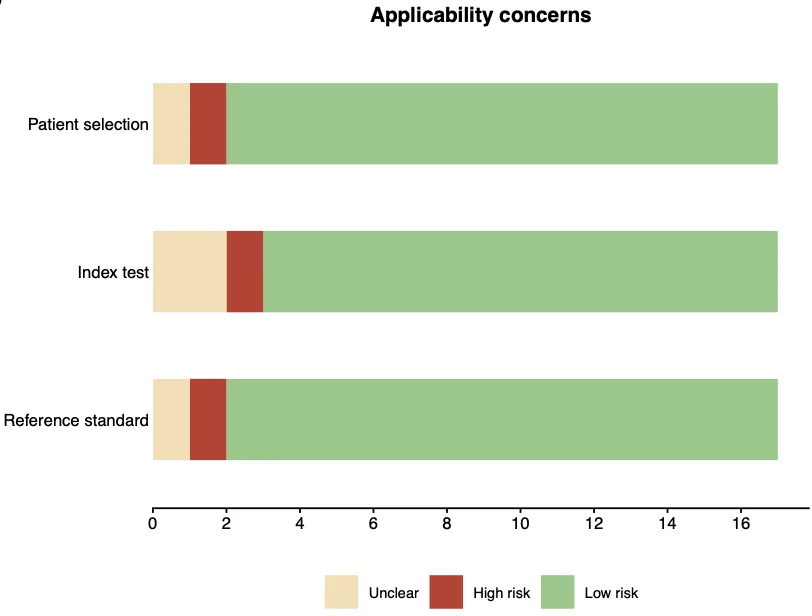
Applicability concerns across included studies.

Risk-of-bias assessment using the QUADAS-2 tool is summarized in Figures 3-5. Most studies were rated as low risk across all domains; however, unclear or high risk of bias remained common, particularly in the index test domain, where 47.1% (8/17) and 23.5% (4/17) of studies were rated as unclear and high risk, respectively. High risk of bias was also identified in patient selection (5.8%; 1/17) and flow and timing (11.8%; 2/17). Applicability concerns were generally low, although isolated studies demonstrated high or unclear concern in patient selection, index test, and reference standard domains.

GRADE assessment (Table 3) rated the certainty of evidence for the pooled sensitivity and specificity as low, primarily because of study limitations identified by QUADAS-2 and substantial between-study heterogeneity. No serious concerns were identified regarding indirectness, imprecision, or publication bias.

**Table 3.** Certainty of evidence for the pooled diagnostic accuracy outcomes assessed using the GRADE^a^ approach for diagnostic test accuracy, considering risk of bias, inconsistency, indirectness, imprecision, and publication bias.

Outcome	Studies	Participant (n)	Risk of bias	Inconsistency	Indirectness	Imprecision	Publication bias	Overall certainty
Overall pooled sensitivity	17	11542^b^	Serious	Serious	Not serious	Not serious	Undetected	⊕⊕○○^c^
Overall pooled specificity	17	11542^b^	Serious	Serious	Not serious	Not serious	Undetected	**⊕⊕○○**

a GRADE: Grading of Recommendations Assessment, Development and Evaluation.

b One included study did not report the number of participants contributing to the diagnostic accuracy analysis; therefore, the total number of included participants may be slightly underestimated.

c ⊕⊕◯◯: low certainity.

### Diagnostic Performance

**Figure 6.**
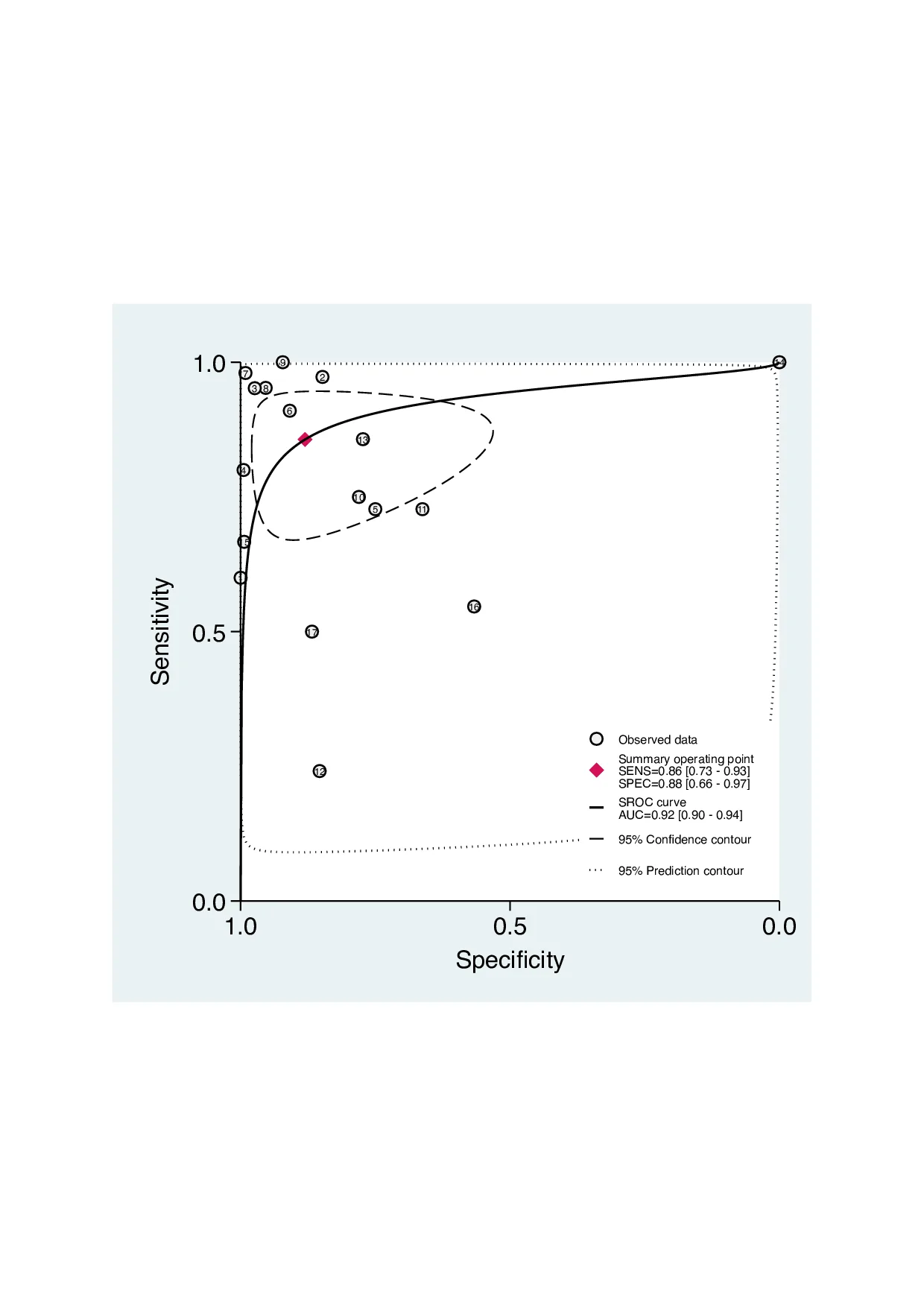
Overall diagnostic performance of AI–based models for prediction and detection of compromised microvascular free flaps across 17 included studies. Summary receiver operating characteristic (SROC) curve demonstrating pooled diagnostic accuracy across image-based and clinical-variable–based models. Study labels correspond to the following studies: (1): Zhang et al, 2026 [25]; (2): He et al, 2026 [26]; (3): Huang et al, 2026 [27]; (4): Kim et al, 2026 [58]; (5): Maktabi et al, 2025 [55]; (6): Kim et al, 2024 [46]; (7): Huang et al, 2023 [47]; (8): Hsu et al, 2023 [48]; (9): Monarchi et al, 2025 [56]; (10): Oleru et al, 2025 [54]; (11): Fang et al, 2025 [57]; (12): Wang et al, 2024 [49]; (13): Yang et al, 2024 [50]; (14): Asaad et al, 2023 [51]; (15): Shi et al, 2022 [53]; (16): Tighe et al, 2022 [52]; 17: O’Neil et al, 2020 [20]. AUC: area under the curve; SENS: sensitivity; SPEC: specificity.

**Figure 7.**
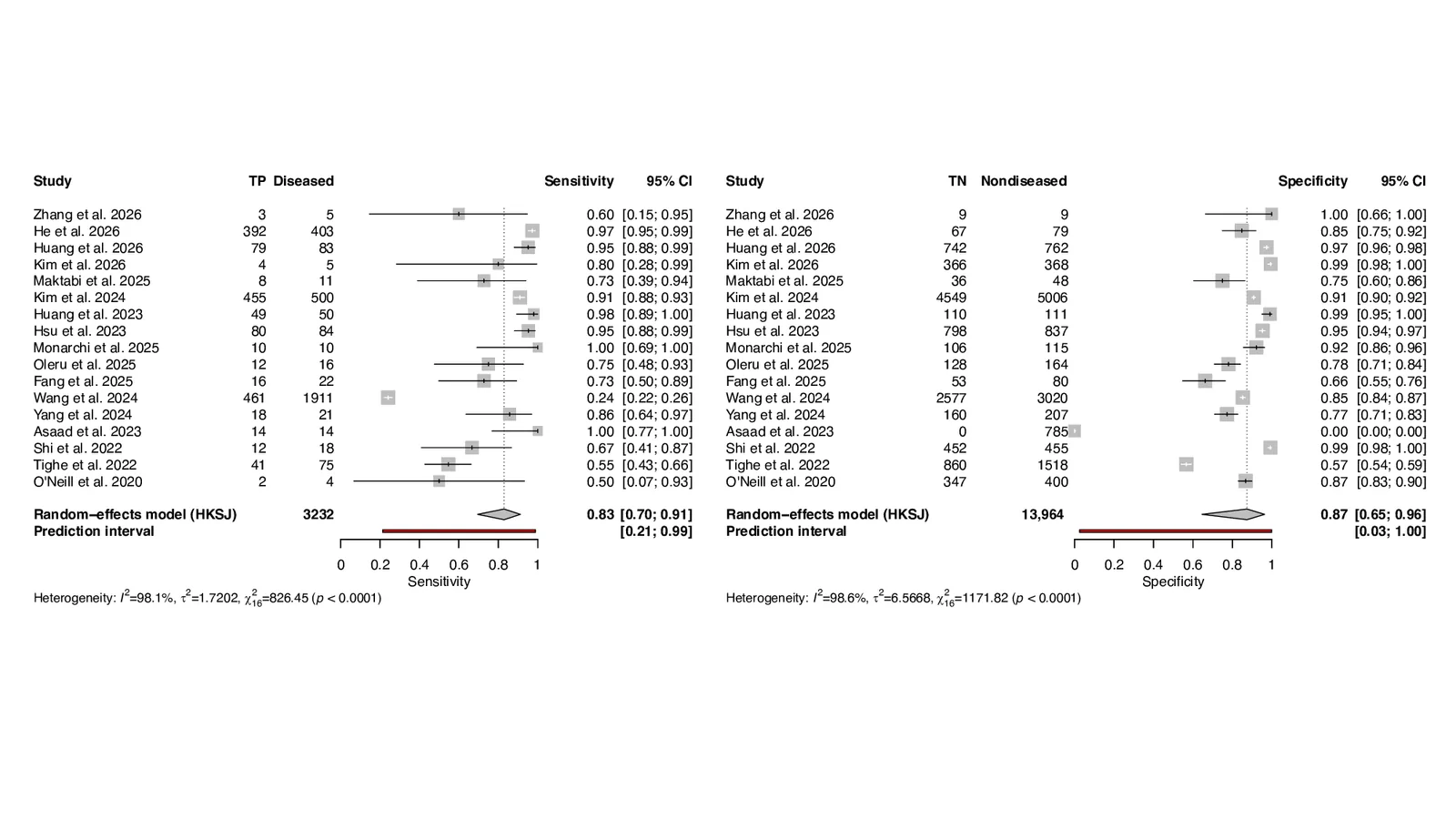
Coupled forest plots of pooled sensitivity and specificity [20,25-27,46-58]. HKSJ: Hartung-Knapp-Sidik-Jonkman; TN: true negative; TP: true positive.

**Figure 8.**
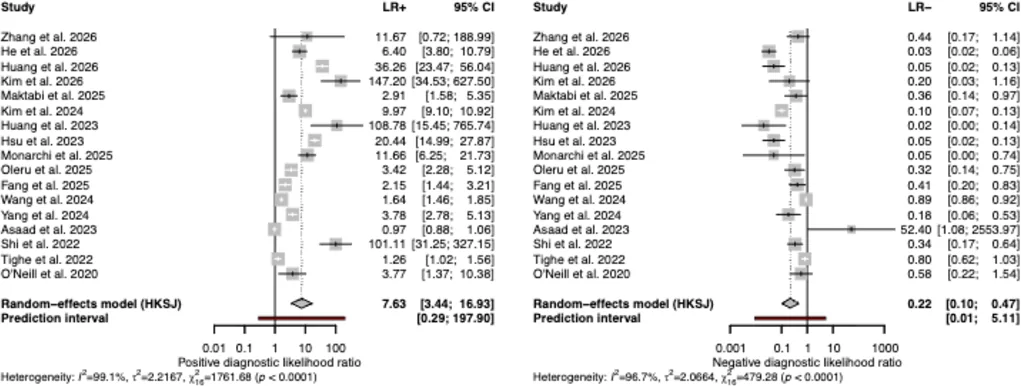
Forest plots of positive likelihood ratio and negative likelihood ratio [20,25-27,46-58]. HKSJ: Hartung-Knapp-Sidik-Jonkman; LR: likelihood ratio.

**Figure 9.**
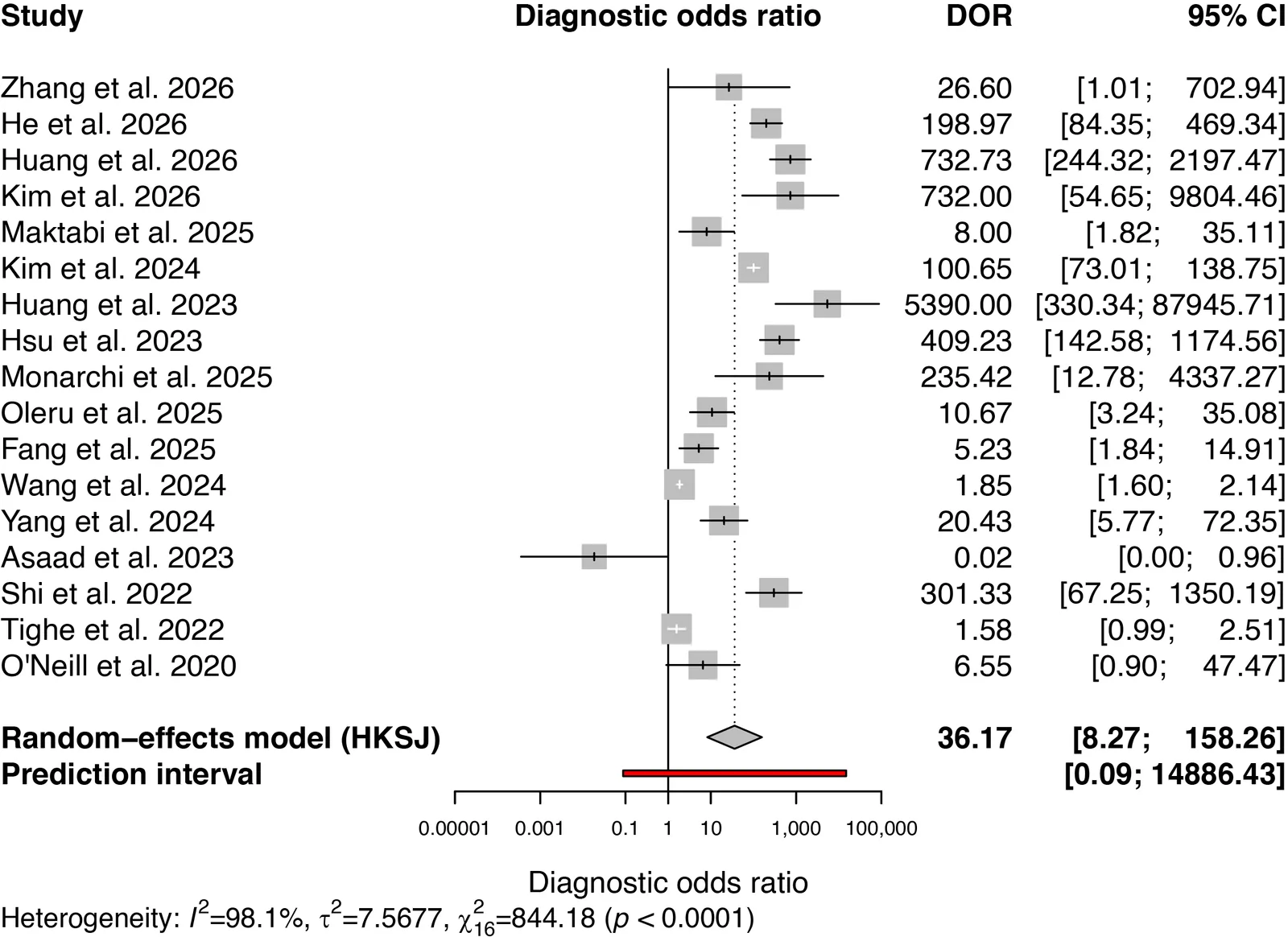
Forest plot of diagnostic odds ratio (DOR). Study labels correspond to the included studies listed in numerical order [20,25-27,46-58]. HKSJ: Hartung-Knapp-Sidik-Jonkman.

Pooled diagnostic accuracy across the 17 studies [20,25,26,46-57] demonstrated an overall sensitivity of 0.83 (95% CI 0.70‐0.91; PI 0.21‐0.99) and a specificity of 0.87 (95% CI 0.65‐0.96; PI 0.03‐1.00). The AUC was 0.92 (95% CI 0.90‐0.94), with a positive likelihood ratio (PLR) of 7.63 (95% CI 3.44‐16.93; PI 0.29‐197.90), a negative likelihood ratio (NLR) of 0.22 (95% CI 0.10‐0.47; PI 0.01‐5.11), and a DOR of 36.17 (95% CI 8.27‐158.26; PI 0.09‐14886.43; Figures 6-9). Study-level DORs varied widely, ranging from 0.02 to 5390.00, largely because DOR estimates are highly sensitive to very small FP or FN counts.

The paired forest plot (Figure 7) showed that image-based models generally achieved higher sensitivity than models based on clinical variables, with 7 studies [26,27,46-48,51,56] reporting sensitivities exceeding 90%. Specificity estimates ranged from 0.00 to 1.00 across the included studies. Substantial between-study heterogeneity was observed for both sensitivity: (*I*²=98.1%) and specificity (*I*²=98.6%), with broad prediction intervals (sensitivity: 0.21‐0.99; specificity: 0.03‐1.00). These findings should be interpreted together with the substantial heterogeneity and the low certainty of evidence (Table 3).

### Clinical Value Analysis

To assess clinical applicability, a Fagan nomogram (Figure S1 in Multimedia Appendix 2) was constructed using a pretest probability of 19%, reflecting the estimated prevalence of flap compromise in the included population. A positive test result increased the posttest probability of flap compromise from 19% to approximately 62%, while a negative test result reduced it to 4%. These findings highlight the strong rule-in and rule-out capabilities of AI-based models, underscoring their potential utility in clinical decision-making for flap monitoring.

### Subgroup Analysis

#### Image-Based Assessment of Flap Viability

**Figure 10.**
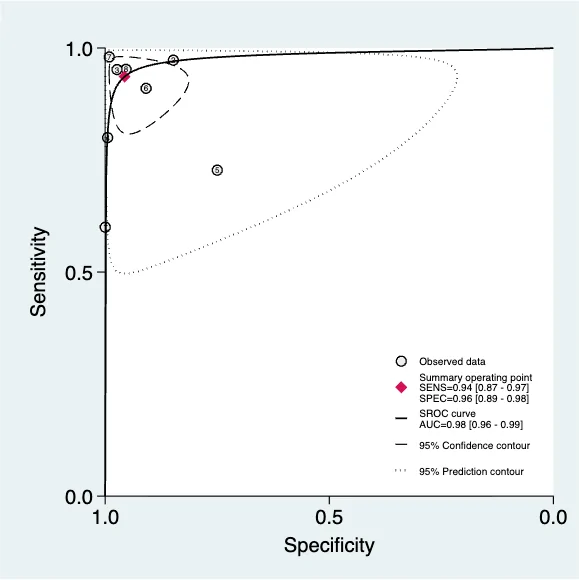
In this subgroup analysis, we examined image-based models developed for dynamic surveillance of flap perfusion. Pooled accuracy was high, including a positive likelihood ratio of 17.85 (95% CI 5.87‐54.31; PI 0.81‐393.30), a negative likelihood ratio of 0.09 (95% CI 0.01‐1.17), and a diagnostic odds ratio of 206.05 (95% CI 40.65‐1044.49; PI 2.29‐18579.34). Summary receiver operating characteristic curve. Study labels correspond to the following studies: (1): Zhang et al, 2026 [25]; (2): He et al, 2026 [26]; (3): Huang et al, 2026 [27]; (4): Kim et al, 2026 [58]; (5): Maktabi et al, 2025 [55]; (6): Kim et al, 2024 [46]; (7): Huang et al, 2023 [47]; (8): Hsu et al, 2023 [48]. AUC: area under the curve; SENS: sensitivity; SPEC: specificity.

**Figure 11.**
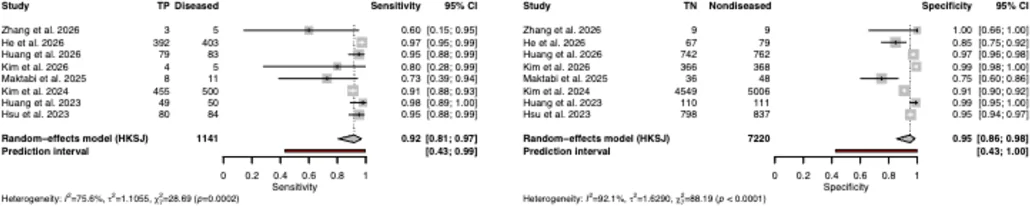
Forest plot of sensitivity and specificity [25-27,46-48,55,58]. HKSJ: Hartung-Knapp-Sidik-Jonkman; TN: true negative; TP: true positive.

Subgroup analyses revealed notable differences between methodological approaches. Pooled analysis of the 8 image-based studies [25-27,46-48,55,58] demonstrated a sensitivity of 0.92 (95% CI 0.81‐0.97; PI 0.43‐0.99) and a specificity of 0.95 (95% CI 0.86‐0.98; PI 0.43‐1.00), with an AUC of 0.98 (95% CI 0.96‐0.99) and a DOR of 206.05 (95% CI 40.65‐1044.49; PI: 2.29‐18579.34), confirming robust diagnostic accuracy (Figure 10). Visual inspection of the forest plot demonstrated consistently high performance across most studies, with sensitivity and specificity estimates exceeding 0.90 (Figure 11). Zhang et al [25] reported the lowest sensitivity (0.60, 95% CI 0.15‐0.95), whereas Maktabi et al [55] demonstrated the lowest specificity (0.75, 95% CI 0.60‐0.86).

#### Performance of Clinical Variable-Based Models

**Figure 12.**
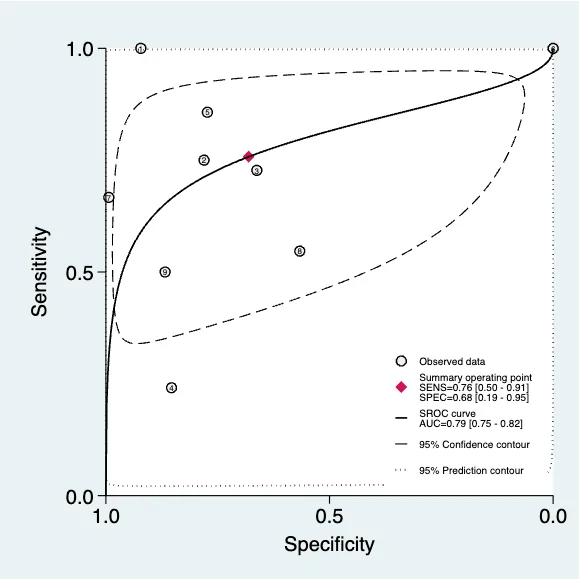
In this subgroup analysis, we examined clinical variable-based models developed for dynamic surveillance of flap perfusion. Pooled accuracy was moderate, including a positive likelihood ratio of 3.72 (95% CI 1.30‐10.65; PI 0.14‐96.31), a negative likelihood ratio of 0.48 (95% CI 0.17‐1.32; PI 0.02‐13.72), and a diagnostic odds ratio of 7.64 (95% CI 1.01‐57.57; PI 0.02‐3623.04). Summary receiver operating characteristic curve. Study labels correspond to the following studies: (1): Monarchi et al, 2025 [56]; (2): Oleru et al, 2025 [54]; (3): Fang et al, 2025 [57]; (4): Wang et al, 2024 [49]; (5): Yang et al, 2024 [50]; (6): Asaad et al, 2023 [51]; (7): Shi et al, 2022 [53]; (8): Tighe et al, 2022 [52]; (9): O’Neil et al, 2020 [20]. AUC: area under the curve; SENS: sensitivity; SPEC: specificity.

Pooled analysis of the 9 studies [20,49-54,56,57] relying on clinical variables demonstrated more modest performance, with a sensitivity of 0.69 (95% CI 0.45‐0.86; PI 0.11‐0.98) and a specificity of 0.72 (95% CI 0.18‐0.97; PI 0.00‐1.00). The corresponding AUC was 0.79 (95% CI 0.75‐0.82) (Figure 12), and the DOR was 7.64 (95% CI 1.01‐57.57; PI 0.02‐3623.04), indicating only moderate discriminative ability.

**Figure 13.**
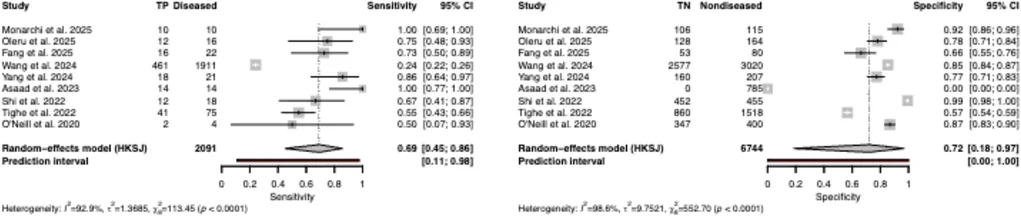
Forest plot of sensitivity and specificity [20,49-54,56,57]. HKSJ: Hartung-Knapp-Sidik-Jonkman; TN: true negative; TP: true positive.

Variability was evident in the paired forest plot (Figure 13), with Wang et al [49] reporting the very lowest sensitivity (0.24, 95% CI 0.22‐0.26), whereas Asaad et al [51] demonstrated an extreme specificity of 0.00 (95% CI 0.00‐0.00).

#### Prediction and Detection of Vascular Compromise

**Figure 14.**
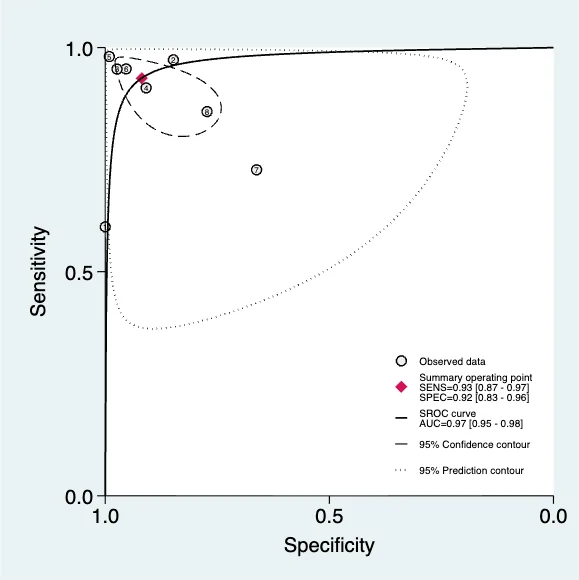
For the subgroup of AI models targeting vascular compromise, overall diagnostic performance was synthesized and visualized using summary receiver operating characteristic (SROC) curves and forest plots. The pooled analysis revealed a high level of accuracy, with a positive likelihood ratio of 10.71 (95% CI 3.90-29.42; PI 0.63‐183.47) and a negative likelihood ratio of 0.10 (95% CI 0.04-0.25; PI 0.01‐1.33). This corresponds to a summary diagnostic odds ratio of 124.23 (95% CI 20.76‐743.41; PI 0.79‐19648.96). Summary receiver operating characteristic curve. Study labels correspond to the following studies: (1): Zhang et al, 2026 [25]; (2): He et al, 2026 [26]; (3): Huang et al, 2026 [27]; (4): Kim et al, 2024 [46]; (5): Huang et al, 2023 [47]; (6): Hsu et al, 2023 [48]; (7): Fang et al, 2025 [57]; (8): Yang et al, 2024 [50]. AUC: area under the curve; SENS: sensitivity; SPEC: specificity.

**Figure 15.**
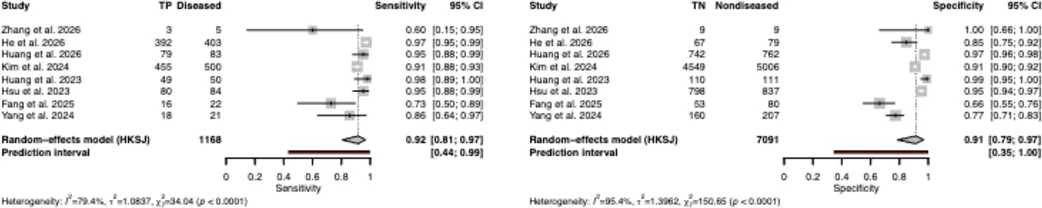
Forest plot of sensitivity and specificity [25-27,46-48,50,57]. HKSJ: Hartung-Knapp-Sidik-Jonkman; TN: true negative; TP: true positive.

The pooled analysis of 8 studies [25-27,46-48,50,57] specifically targeting the prediction and detection of vascular crisis demonstrated strong overall diagnostic accuracy. The model yielded a pooled sensitivity of 0.92 (95% CI 0.81‐0.97; PI 0.44‐0.99) and a specificity of 0.91 (95% CI 0.79‐0.97; PI 0.35‐1.00; Figures 14 and 15). This robust performance was further reflected in an AUC of 0.97 (95% CI 0.95‐0.98; Figure 14) and a DOR of 124.23 (95% CI 20.76‐743.41), indicating satisfactory discriminatory ability for predicting vascular compromised flaps. The paired forest plot (Figure 15) demonstrated high heterogeneity in both sensitivity (*I*^2^=79.4%) and specificity (*I*^2^=95.4%). Zhang et al [25] appeared to contribute disproportionately to the observed variability, reporting the lowest sensitivity and the widest CIs for both sensitivity and specificity, suggesting reduced estimate precision.

### Sensitivity Analysis

To investigate the sources of heterogeneity and assess the robustness of the findings, a comprehensive sensitivity analysis was performed. Initial inspection of the bivariate boxplot suggested that 3 studies [27,51,53] lay outside the main data cluster, raising concerns about its influence (Figure S2a in Multimedia Appendix 3). This prompted a more rigorous statistical evaluation using multiple diagnostic tools. Goodness of fit and bivariate normality were assessed using Q-Q plots based on deviance residuals and Mahalanobis distances, respectively (Figure S2b and S2c in Multimedia Appendix 3). Additional influence analysis using Cook distance (Figure S2d in Multimedia Appendix 3) and outlier detection based on standardized residuals from the bivariate Reitsma model (Figure S2e in Multimedia Appendix 3) were also undertaken. A conservative threshold of > |2.0| for standardized residuals was applied to identify outliers. These diagnostics identified 2 studies of concern, mainly based on the results of influence analysis. Asaad et al [51] was identified as a statistical outlier (Figure S2e in Multimedia Appendix 3), while both Asaad et al [51] and Wang et al [49] were shown to exert a disproportionate influence on the pooled estimates (Figure S2d in Multimedia Appendix 3).

**Figure 16.**
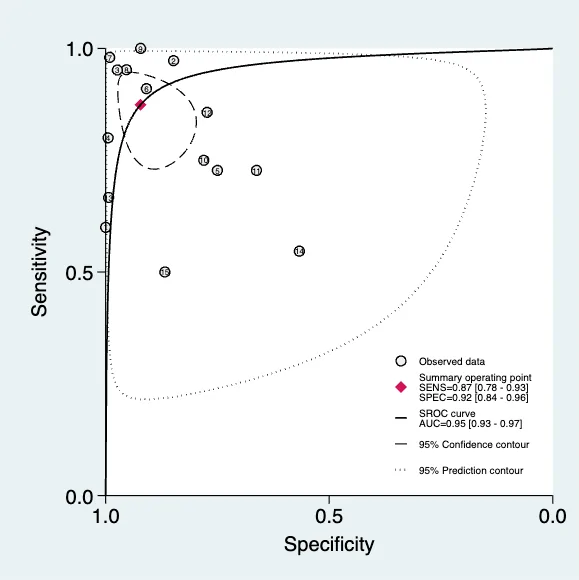
In sensitivity analyses, the outlying studies Wang et al, 2024 [49], and Asaad et al, 2023 [51], were excluded, and the bivariate model was refitted to assess the effect on summary accuracy estimates and between-study heterogeneity (*I*²). Summary receiver operating characteristic curve. Study labels correspond to the following studies: (1): Zhang et al, 2026 [25]; (2): He et al, 2026 [26]; (3): Huang et al, 2026 [27]; (4): Kim et al, 2026 [58]; (5): Maktabi et al, 2025 [55]; (6): Kim et al, 2024 [46]; (7): Huang et al, 2023 [47]; (8): Hsu et al, 2023 [48]; (9): Monarchi et al, 2025 [56]; (10): Oleru et al, 2025 [54]; (11): Fang et al, 2025 [57]; (12): Yang et al, 2024 [50]; (13): Shi et al, 2022 [53]; (14): Tighe et al, 2022 [52]; and (15): O’Neill et al, 2020 [20].

**Figure 17.**
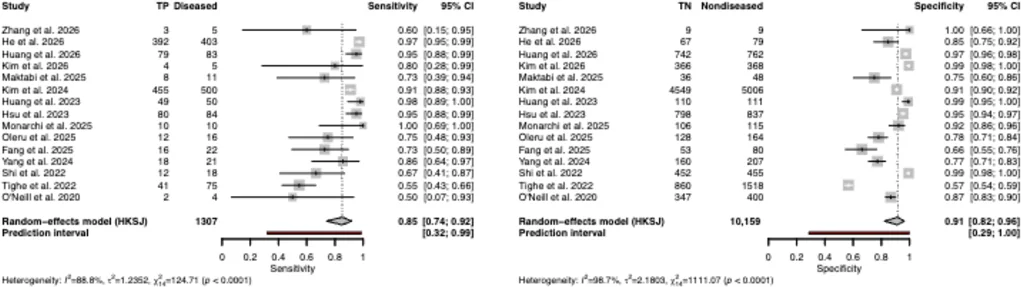
Forest plot of sensitivity and specificity [20,25-27,46-48,50,52-58]. HKSJ: Hartung-Knapp-Sidik-Jonkman; TN: true negative; TP: true positive.

**Figure 18.**
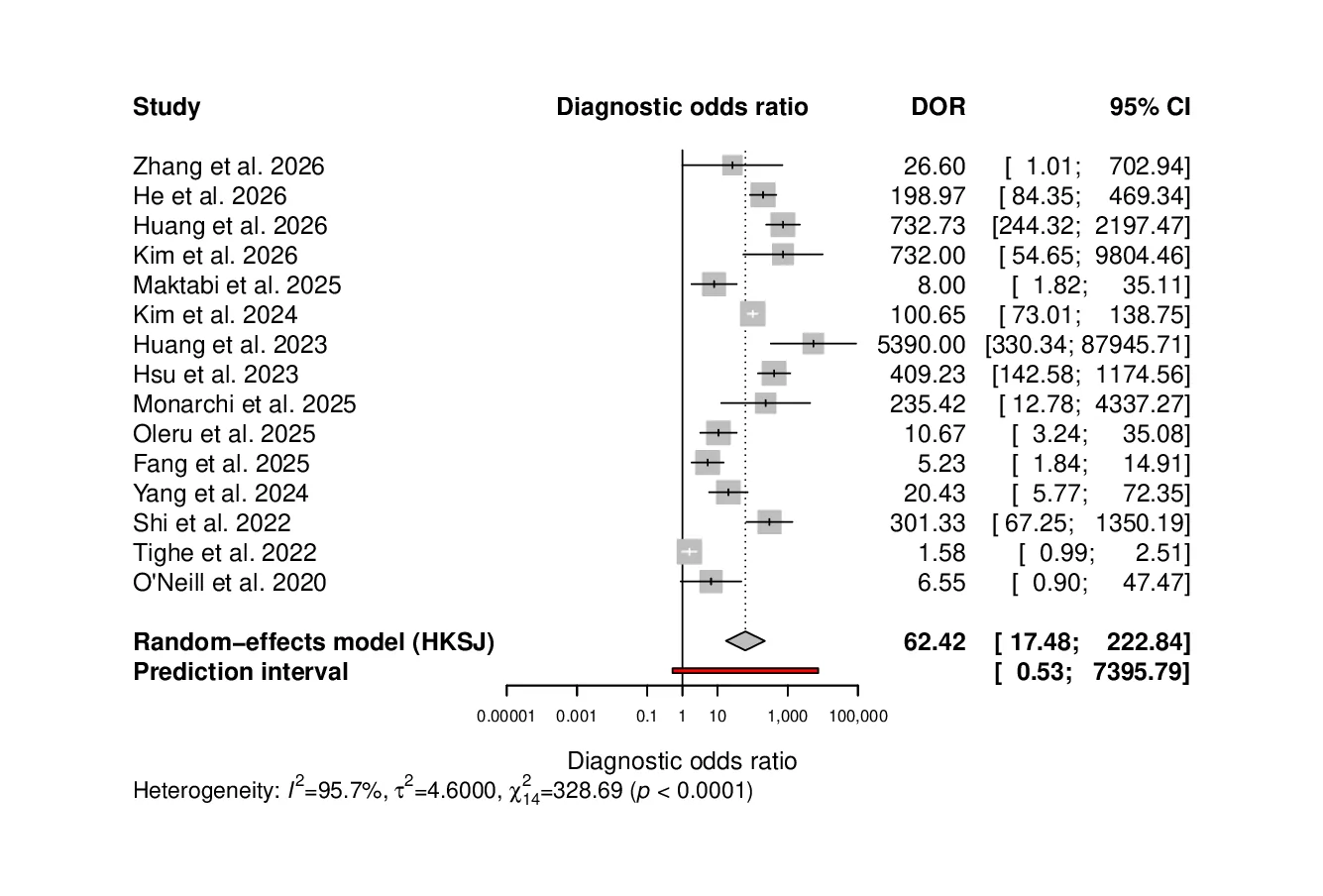
Forest plots of positive likelihood ratio and negative likelihood ratio [20,25-27,46-48,50,52-58]. HKSJ: Hartung-Knapp-Sidik-Jonkman; LR: likelihood ratio.

**Figure 19.**
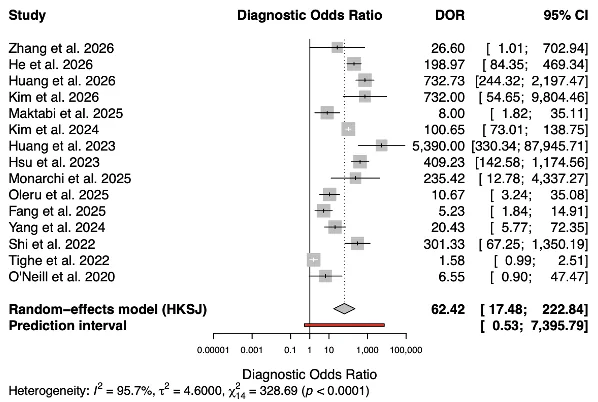
Forest plot of diagnostic odds ratio (DOR) [20,25-27,46-48,50,52-58]. HKSJ: Hartung-Knapp-Sidik-Jonkman.

To evaluate their impact, the sensitivity analysis was performed after excluding Wang et al [49] and Asaad et al [51]. The exclusion did not materially change the summary estimates; both the *I*^2^ and the AUC of the SROC remained stable (Figures 16-19). Since no other studies exceeded the predefined thresholds, all remaining studies were retained in the final model. Collectively, these findings confirm that the overall results are robust and not unduly influenced by any single study, thereby reinforcing the validity of the pooled conclusions.

### Small-Study Effects

Small study effects were assessed using Deeks funnel plot asymmetry test (Figure S3 in Multimedia Appendix 4). The analysis revealed no significant funnel plot asymmetry was observed (*P*=.15>.10), indicating no evidence of small-study effects.

## Discussion

### Principal Findings

This systematic review and meta-analysis provides a comprehensive evaluation of AI–based algorithms for postoperative surveillance of microvascular free flaps. Overall, image-based approaches demonstrated favorable diagnostic performance and generally outperformed models based solely on clinical variables, supporting the potential role of AI-assisted monitoring in microsurgical care.

Timely detection of vascular compromise remains fundamental to successful free flap salvage [9]. Although overall flap success exceeds 95%, vascular compromise remains one of the leading causes of flap loss and frequently necessitates urgent reexploration [1,9]. Current postoperative monitoring relies heavily on serial clinical examination, making assessment susceptible to interobserver variability, fatigue, and subjective interpretation [4,12]. Several adjunctive monitoring technologies, including implantable Doppler devices, tissue oximetry, near-infrared spectroscopy, and indocyanine green angiography, have improved flap surveillance, but remain limited by cost, workflow integration, equipment availability, and the absence of standardized protocols [2,13,60,61].

AI offers a complementary strategy by providing continuous and objective interpretation of clinical or imaging data [22]. In addition to postoperative monitoring, AI-based prediction models may facilitate preoperative risk stratification, enabling individualized perioperative planning and more efficient allocation of monitoring resources [14]. In this study, models incorporating photographic or image-based inputs consistently outperformed those based solely on clinical variables. This finding is biologically plausible because visual information directly reflects dynamic physiological changes, including alterations in flap color, congestion, and tissue perfusion, that may precede overt clinical deterioration and cannot be fully captured by conventional clinical variables alone [62].

Studies by Huang et al and Maktabi et al further support this concept using RGB color analysis and hyperspectral imaging–derived physiologic parameters [47,55]. To better reflect clinical applicability, data from Huang et al [27] were extracted from an independent clinical validation cohort rather than the training cohort, thereby providing an estimate that more closely represents real-world diagnostic performance. Recent advances also illustrate the rapid evolution of this field. Huang et al [47] employed a Random Forest model [63], Kim et al [46] implemented a DenseNet121 deep learning architecture using TensorFlow (Google LLC) in Python 3.7 (Python Software Foundation) [64], Hsu et al [48] introduced the smartphone-integrated “FLAPMATE” platform for real-time postoperative monitoring, and Huang et al [27] presented the “DLscope” system, which integrates a camera device, data transmission, server, and smartphone application to support remote flap surveillance. Collectively, these developments highlight the increasing feasibility of incorporating AI-assisted assessment into routine microsurgical practice [27,48,63].

In contrast, models that relied exclusively on clinical variables achieved more modest performance, likely reflecting both the multifactorial nature of flap compromise and heterogeneity in predictor selection across studies [20,49-54,56,57]. However, these models may still provide practical value for risk stratification. By categorizing patients into high and low risk tiers, these models may help guide the intensity of postoperative surveillance and allocation of clinical resources. Frequently identified predictors, including smoking, female sex, and elevated BMI, are well-recognized clinical risk factors, suggesting that the strength of these models lies in integrating multiple modest predictors into an individualized estimate of postoperative flap-related risk [65-67].

Despite their promising diagnostic performance, several factors currently limit the translation of AI into routine microsurgical practice [68]. Image-based algorithms remain susceptible to variations in lighting conditions, camera systems, skin pigmentation, wound dressings, surgical drains, and postoperative edema, all of which may influence image quality and model performance [68,69]. In routine postoperative settings, image quality may also be influenced by edema, dried blood, dressings, surgical drains, or Doppler wires [69]. Moreover, clinically important findings such as early venous congestion may present with only subtle visual changes, making robust model generalization across institutions and patient populations particularly challenging [55]. These observations highlight the importance of prospective multicenter validation and standardized image acquisition before widespread clinical implementation [68].

Substantial clinical and methodological heterogeneity was observed across the included studies [20,25,26,46-57]. Outcome definitions ranged from vascular compromise requiring reexploration to complete flap failure, while AI approaches varied from conventional machine-learning algorithms to deep learning-based image analysis. Considerable differences were also observed in predictor selection, sample size, event rates, and the timing of model application, with studies evaluating both preoperative risk prediction and postoperative flap surveillance [45]. These variations likely contributed to the substantial heterogeneity observed in the pooled analyses.

The wide variation in study-level DORs should not be interpreted solely as indicating intrinsic superiority of certain AI models. Because DOR is highly sensitive to small FP or FN counts, studies with few misclassifications may produce very large estimates with wide CIs [70]. Differences in populations, outcome definitions, AI algorithms, decision thresholds, validation methods, and class distributions may have further contributed to this variability. Importantly, the pooled sensitivity, specificity, and DOR should be interpreted as estimates of the average diagnostic performance across the included studies. Although the CIs describe the uncertainty around these pooled average estimates, the wide PIs demonstrate that diagnostic performance may vary substantially across different clinical settings [45]. Therefore, the pooled estimates should not be interpreted as performance that can be uniformly expected in all practice environments.

The methodological assessment also supports cautious interpretation of these findings. QUADAS-2 identified concerns regarding patient selection and index test methodology in several studies [71], and the GRADE assessment rated the certainty of evidence as low because of study limitations and substantial between-study heterogeneity [72]. Although the pooled estimates suggest favorable diagnostic performance, the low certainty of evidence according to GRADE indicates that these findings should be interpreted cautiously and may not be readily generalizable across different clinical settings. Accordingly, the current evidence supports the potential of AI-assisted monitoring but remains insufficient to justify widespread clinical implementation without prospective multicenter validation and rigorous external evaluation.

Another important methodological consideration relates to model development and reporting quality [73]. Flap compromise was an infrequent event in many included datasets, resulting in substantial class imbalance [74]. Under these conditions, models may achieve high specificity and AUC despite limited sensitivity for detecting uncommon adverse events [75]. This phenomenon was evident in several studies, particularly those with very low event rates, and may have contributed to optimistic estimates of diagnostic performance [56]. Although some investigators adopted imbalance-correction techniques, including data augmentation or synthetic oversampling, these approaches were inconsistently applied across studies and may have contributed to the observed between-study heterogeneity [73,76].

Several limitations should be acknowledged. Most included studies were retrospective, single-center investigations with limited external validation, increasing the risk of selection bias and model overfitting [77,78]. In addition, several studies demonstrated unclear or high risk of bias in the patient selection and index test domains, primarily related to limited reporting transparency and lack of external validation. Although sensitivity analyses demonstrated relative stability of pooled estimates, these methodological limitations should be considered when interpreting the findings.

To ensure consistent quantitative synthesis, several methodological decisions were required during data extraction. When multiple AI algorithms were reported within a study, only the best-performing model was included because it most closely represents the model likely to be translated into clinical practice. Although this approach may slightly overestimate diagnostic performance, including multiple models derived from the same cohort would have disproportionately weighted individual datasets and violated the assumption of independent observations [78]. Furthermore, prioritizing higher sensitivity as a secondary tie-breaking criterion may increase FP alerts and clinical workload, but this situation occurred in only one included study because AUC alone determined model selection in the remaining studies [68]. Therefore, the impact of this selection strategy on the pooled estimates was likely limited.

Outcome definitions also required harmonization across studies. For example, Kim et al [58] originally classified postoperative outcomes as confirmed compromised, suspicious, and normal flaps. To maintain consistency with diagnostic test accuracy methodology, suspicious and normal flaps were combined into a single non-compromised category before reconstruction of the 2×2 contingency table. Similarly, several studies did not report TP, FP, TN, and FN directly, requiring reconstruction from published diagnostic performance measures [47,51,53-57]. Although this approach is widely accepted in diagnostic accuracy meta-analyses, it may have introduced additional uncertainty into the pooled estimates [33].

Crucially, the diagnostic fidelity of these AI systems remains inherently tethered to the “silver standard” of current microsurgical practice [68,78]. Given that the ground truth often relies on subjective clinical intuition, characterized by an inherent inter-observer variability, the reported diagnostic performance may represent a theoretical ceiling imposed by the noise within training labels [68]. Furthermore, the necessity of post hoc data reconstruction for 44% (8/18) of the included studies suggests a pervasive reporting gap in the microsurgical literature [78].

This review provides the most comprehensive synthesis of diagnostic test accuracy evidence currently available for AI in postoperative free flap monitoring [23,24]. Unlike previous systematic reviews, this review adopts a clinically oriented approach by focusing on postoperative free flap monitoring rather than postoperative complication prediction alone. It further distinguishes image-based surveillance from clinical variable-based risk stratification, providing evidence for their complementary roles in postoperative free flap care. By applying established diagnostic test accuracy methodology, this review provides a robust evaluation of AI-assisted detection of free flap compromise. It further demonstrates how different AI approaches may complement postoperative care, with image-based algorithms assisting diagnosis and clinical variable-based models enabling individualized risk stratification and more efficient allocation of monitoring resources.

Collectively, these findings suggest that AI may contribute to postoperative free flap care in multiple ways. Image-based algorithms demonstrated strong diagnostic performance for early detection of vascular compromise and may enhance real-time postoperative surveillance through objective and reproducible assessment. In contrast, models based on clinical variables may provide additional value for perioperative risk stratification, enabling individualized surveillance strategies and more efficient allocation of monitoring resources. Together, these approaches have the potential to improve the consistency of flap monitoring, facilitate timely clinical decision-making, and complement rather than replace clinical judgment. Before routine clinical implementation, prospective multicenter studies with standardized imaging protocols, rigorous external validation, and transparent reporting in accordance with Standards for Reporting of Diagnostic Accuracy Studies-AI (STARD-AI) and decision support systems driven by artificial intelligence (DECIDE-AI) are needed to establish generalizability, support regulatory evaluation, and ensure the safe and responsible integration of AI into microsurgical practice [79-82].

### Conclusion

AI-driven flap surveillance, particularly through image-based deep learning, demonstrates strong diagnostic performance. Its application may facilitate earlier detection, reduce diagnostic errors, and support postoperative monitoring in both hospital and remote settings. However, these findings should be interpreted cautiously because several studies were based on relatively small retrospective datasets with limited external validation. Broader clinical adoption will require further high-quality prospective validation and standardized reporting, and these technologies should be integrated into clinical practice as adjuncts to, rather than replacements for, surgical judgment.

## Supplementary material

10.2196/91174 Multimedia Appendix 1 Five databases (PubMed, Embase, Cochrane Library, Web of Science, and Scopus) were searched systematically with keywords as follows: Free flap assessment, artificial intelligence, diagnostic accuracy.

10.2196/91174 Multimedia Appendix 2 Fagan nomogram demonstrating the posttest probability and potential clinical utility of AI–based models for prediction and detection of flap compromise in free flap reconstruction.

10.2196/91174 Multimedia Appendix 3 Sensitivity and influence analyses of pooled diagnostic performance across studies evaluating AI–based models for free flap surveillance.

10.2196/91174 Multimedia Appendix 4 Deeks funnel plot asymmetry test evaluating potential publication bias among studies assessing AI–based diagnostic models for free flap compromise.

10.2196/91174 Checklist 1 PRISMA-S checklist.

10.2196/91174 Checklist 2 PRISMA 2020 checklist.

## Abbreviations

ALT: anterolateral thigh
AUC: area under the curve
DECIDE-AI: decision support systems driven by artificial intelligence
DOR: Diagnostic odds ratios
FN: false negative
FP: false positive
GRADE: Grading of Recommendations Assessment, Development, and Evaluation
NLR: negative likelihood ratio
PI: prediction interval
PLR: positive likelihood ratio
PRISMA: Preferred Reporting Items for Systematic Reviews and Meta-Analyses
PRISMA-DTA: Preferred Reporting Items for Systematic Reviews and Meta-Analyses of Diagnostic Test Accuracy Studies
PRISMA-S: Preferred Reporting Items for Systematic Reviews and Meta-Analyses literature search extension
PROSPERO: International Prospective Register of Systematic Reviews
QUADAS-2: Quality Assessment of Diagnostic Accuracy Studies-2
SROC: summary receiver operating characteristic
STARD-AI: Standards for Reporting of Diagnostic Accuracy Studies-AI
TITAN: Transparency in the reporting of AI
TN: true negative
TP: true positive
