# Supplementary material for: Diagnostic Accuracy of AI in Prediction and Assessment of Compromised Free Flaps: Systematic Review and Meta-Analysis
Source: J Med Internet Res. 2026 Sep 22;28:e91174. doi: 10.2196/91174 (PMC13596764; doi:10.2196/91174)
Supplement: Supplementary file 2 [file jmir-v28-e91174-s001.docx]

**TABLE S1**

The following material accompanies the article **“Accuracy of Artificial Intelligence in Prediction and Detection of Compromised Free Flaps: A Systematic Review and Meta-Analysis”**

**Table S1: Electronic search strategy**

Five databases (PubMed, Embase, Cochrane Library, Web of science, and Scopus) were searched systematically with keywords as follows: Free flap assessment, artificial intelligence, diagnostic accuracy.

**Table S1** Electronic search strategy

**Search time:** 2026/06/21

Database: Pubmed、Cochrane、Embase、Web of science、Scopus

1. **Artificial intelligence**

Prediction model [Title/Abstract]

Artificial intelligence [Title/Abstract]

Deep learning [Title/Abstract]

Machine learning [Title/Abstract]

Neural network [Title/Abstract]

Computer vision [Title/Abstract]

Convolutional neural network [Title/Abstract]

CNN [Title/Abstract]

Radiomics [Title/Abstract]

AI-driven prediction [Title/Abstract]

Automated diagnosis [Title/Abstract]

Predictive analytics [Title/Abstract]

Algorithm development [Title/Abstract]

Data-driven model [Title/Abstract]

Prediction [Title/Abstract]

Risk prediction [Title/Abstract]

Risk model [Title/Abstract]

Diagnostic model [Title/Abstract]

Decision support [Title/Abstract]

Computer-aided diagnosis [Title/Abstract]

Artificial neural network [Title/Abstract]

Clinical prediction model [Title/Abstract]

Predictive model [Title/Abstract]

Image analysis [Title/Abstract]

Image classification [Title/Abstract]

Feature extraction [Title/Abstract]

Pattern recognition [Title/Abstract]

Algorithm [Title/Abstract]

Clinical decision support [Title/Abstract]

2. **Flap condition**

Flap survival [Title/Abstract]

Flap necrosis [Title/Abstract]

Free flap reconstruction [Title/Abstract]

Free flap monitoring [Title/Abstract]

Flap failure [Title/Abstract]

Flap perfusion [Title/Abstract]

Microvascular flap [Title/Abstract]

Ischemic flap [Title/Abstract]

Surgical flap [Title/Abstract]

Postoperative flap monitoring [Title/Abstract]

Flap viability [Title/Abstract]

Flap compromised [Title/Abstract]

Flap compromise [Title/Abstract]

Compromised circulation [Title/Abstract]

Flap ischemia [Title/Abstract]

Partial flap loss [Title/Abstract]

Total flap loss [Title/Abstract]

Flap congestion [Title/Abstract]

Venous congestion [Title/Abstract]

Arterial insufficiency [Title/Abstract]

Flap monitoring device [Title/Abstract]

Free flap [Title/Abstract]

Free tissue transfer [Title/Abstract]

Free tissue reconstruction [Title/Abstract]

Microsurgery [Title/Abstract]

Microsurgical reconstruction [Title/Abstract]

Reconstructive microsurgery [Title/Abstract]

Microvascular reconstruction [Title/Abstract]

Perforator flap [Title/Abstract]

Flap reconstruction [Title/Abstract]

Vascular compromise [Title/Abstract]

Vascular crisis [Title/Abstract]

Perfusion monitoring [Title/Abstract]

Ischemic complication [Title/Abstract]

Compromised flap [Title/Abstract]

Flap salvage [Title/Abstract]

Re-exploration [Title/Abstract]

Take-back [Title/Abstract]

Takeback [Title/Abstract]

Postoperative monitoring [Title/Abstract]

| Database | Search strategy |
| --- | --- |
| **PubMed**  **(259 studies)** | ("Artificial Intelligence"[Mesh] OR "Machine Learning"[Mesh] OR artificial intelligence[tiab] OR AI[tiab] OR machine learning[tiab] OR deep learning[tiab] OR neural network*[tiab] OR convolutional neural network*[tiab] OR CNN[tiab] OR computer vision[tiab] OR radiomic*[tiab] OR image analysis[tiab] OR image classification[tiab] OR feature extraction[tiab] OR algorithm*[tiab] OR prediction model*[tiab] OR predictive model*[tiab] OR risk model*[tiab] OR diagnostic model*[tiab] OR predictive analytics[tiab] OR decision support[tiab] OR computer-aided diagnosis[tiab] OR automated diagnosis[tiab] OR data-driven model*[tiab]) AND ("Surgical Flaps"[Mesh] OR "Microsurgery"[Mesh] OR free flap*[tiab] OR free tissue transfer[tiab] OR free tissue reconstruction[tiab] OR microvascular flap*[tiab] OR microsurgical reconstruction[tiab] OR reconstructive microsurgery[tiab] OR microvascular reconstruction[tiab] OR perforator flap*[tiab] OR flap reconstruction[tiab] OR surgical flap*[tiab]) AND (flap monitoring[tiab] OR postoperative monitoring[tiab] OR flap viability[tiab] OR flap survival[tiab] OR flap failure[tiab] OR flap necrosis[tiab] OR flap compromise[tiab] OR compromised flap[tiab] OR vascular compromise[tiab] OR vascular crisis[tiab] OR flap perfusion[tiab] OR perfusion monitoring[tiab] OR ischemic complication*[tiab] OR flap ischemia[tiab] OR venous congestion[tiab] OR arterial insufficiency[tiab] OR partial flap loss[tiab] OR total flap loss[tiab] OR flap salvage[tiab] OR re-exploration[tiab] OR takeback[tiab] OR take-back[tiab]) |
| **Embase**  **(587 studies)** | #1 'artificial intelligence'/exp OR 'machine learning'/exp OR 'deep learning'/exp OR 'neural network'/exp OR 'computer vision'/exp OR "artificial intelligence":ti,ab OR "machine learning":ti,ab OR "deep learning":ti,ab OR "neural network":ti,ab OR "neural networks":ti,ab OR "artificial neural network":ti,ab OR "convolutional neural network":ti,ab OR "computer vision":ti,ab OR radiomics:ti,ab OR "image analysis":ti,ab OR "image classification":ti,ab OR "feature extraction":ti,ab OR algorithm:ti,ab OR algorithms:ti,ab  #2 'flap monitoring':ab,ti OR 'flap viability':ab,ti OR'flap survival':ab,ti OR 'flap failure':ab,ti OR 'flap necrosis':ab,ti OR 'flap compromise':ab,ti OR 'vascular compromise':ab,ti OR 'vascular crisis':ab,ti OR 'flap perfusion':ab,ti OR 'perfusion monitoring':ab,ti OR 'flap ischemia':ab,ti OR 'venous congestion':ab,ti OR 'arterial insufficiency':ab,ti OR 'partial flap loss':ab,ti OR 'total flap loss':ab,ti OR 'flap salvage':ab,ti OR 're-exploration':ab,ti  #3: #1 AND #2 |
| **Cochrane library**  **(132 studies)** | ("prediction model" OR "artificial intelligence" OR "deep learning" OR "machine learning" OR "neural network" OR "computer vision" OR "convolutional neural network" OR CNN OR radiomics OR "AI-driven prediction" OR "automated diagnosis" OR "predictive analytics" OR "algorithm development" OR "data-driven model" OR prediction OR "risk prediction" OR "risk model" OR "diagnostic model" OR "decision support" OR "computer-aided diagnosis") AND ("flap survival" OR "flap necrosis" OR "free flap reconstruction" OR "free flap monitoring" OR "flap failure" OR "flap perfusion" OR "microvascular flap" OR "ischemic flap" OR "surgical flap" OR "postoperative flap monitoring" OR "flap viability" OR "flap compromised" OR "flap compromise" OR "compromised circulation" OR "flap ischemia" OR "partial flap loss" OR "total flap loss" OR "flap ischemia" OR "flap congestion" OR "venous congestion" OR "arterial insufficiency" OR "flap monitoring device") |
| **Web of Science**  **(733 studies)** | ("artificial intelligence" OR AI OR "machine learning" OR "deep learning" OR "neural network" OR "convolutional neural network" OR CNN OR "computer vision" OR radiomic OR "image analysis" OR "image classification" OR "feature extraction" OR algorithm OR "prediction model" OR "predictive model" OR "risk model" OR "diagnostic model" OR "predictive analytics" OR "decision support" OR "computer-aided diagnosis" OR "automated diagnosis" OR "data-driven model") AND ("flap monitoring" OR "postoperative monitoring" OR "flap viability" OR "flap survival" OR "flap failure" OR "flap necrosis" OR "flap compromise" OR "compromised flap" OR "vascular compromise" OR "vascular crisis" OR "flap perfusion" OR "perfusion monitoring" OR "ischemic complication" OR "flap ischemia" OR "venous congestion" OR "arterial insufficiency" OR "partial flap loss" OR "total flap loss" OR "flap salvage" OR"re-exploration" OR takeback OR "take-back") viability" OR "flap compromised" OR "flap compromise" OR "compromised circulation" OR "flap ischemia" OR "partial flap loss" OR "total flap loss" OR "flap ischemia" OR "flap congestion" OR "venous congestion" OR "arterial insufficiency" OR "flap monitoring device") |
| **Scopus**  **(383 studies)** | ("artificial intelligence" OR AI OR "machine learning" OR "deep learning" OR "neural network*" OR "convolutional neural network*" OR CNN OR "computer vision" OR radiomic* OR "image analysis" OR "image classification" OR "feature extraction" OR algorithm* OR "prediction model*" OR "predictive model*" OR "risk model*" OR "diagnostic model*" OR "predictive analytics" OR "decision support" OR "computer-aided diagnosis" OR "automated diagnosis" OR "data-driven model*") AND ("free flap*" OR "free tissue transfer" OR "free tissue reconstruction" OR "microvascular flap*" OR "microsurgical reconstruction" OR "reconstructive microsurgery" OR "microvascular reconstruction" OR "perforator flap*" OR "flap reconstruction" OR "surgical flap*") AND ("flap monitoring" OR "postoperative monitoring" OR "flap viability" OR "flap survival" OR "flap failure" OR "flap necrosis" OR "flap compromise" OR "compromised flap" OR "vascular compromise" OR "vascular crisis" OR "flap perfusion" OR "perfusion monitoring" OR "ischemic complication*" OR "flap ischemia" OR "venous congestion" OR "arterial insufficiency" OR "partial flap loss" OR "total flap loss" OR "flap salvage" OR "re-exploration" OR takeback OR "take-back") |
