# Supplementary material for: Diagnostic Accuracy of AI in Prediction and Assessment of Compromised Free Flaps: Systematic Review and Meta-Analysis
Source: J Med Internet Res. 2026 Sep 22;28:e91174. doi: 10.2196/91174 (PMC13596764; doi:10.2196/91174)
Supplement: Supplementary file 3 [file jmir-v28-e91174-s002.docx]

**SUPPLEMENTARY CONTENT**

The following material accompanies the article **“Accuracy of Artificial Intelligence in Prediction and Detection of Compromised Free Flaps: A Systematic Review and Meta-Analysis”**

**Figure S1**

Fagan nomogram demonstrating the posttest probability and potential clinical utility of AI–based models for prediction and detection of flap compromise in free flap reconstruction.

**Figure S1** Fagan nomogram
