# Supplementary material for: Diagnostic Accuracy of AI in Prediction and Assessment of Compromised Free Flaps: Systematic Review and Meta-Analysis
Source: J Med Internet Res. 2026 Sep 22;28:e91174. doi: 10.2196/91174 (PMC13596764; doi:10.2196/91174)
Supplement: Supplementary file 4 [file jmir-v28-e91174-s003.docx]

**FIGURE S2**

The following material accompanies the article **“Accuracy of Artificial Intelligence in Prediction and Detection of Compromised Free Flaps: A Systematic Review and Meta-Analysis”**

**Figure S2**

Sensitivity and influence analyses of pooled diagnostic performance across studies evaluating AI–based models for free flap surveillance. (a) Bivariate boxplot assessing study distribution and heterogeneity. (b) Goodness-of-fit analysis. (c) Bivariate normality assessment. (d) Influence analysis identifying studies with disproportionate effects on pooled estimates. (e) Outlier detection analysis. Study labels correspond to the following studies: 1, Zhang et al. 2026; 2, He et al.2026; 3, Huang et al. 2026; 4, Kim et al. 2026; 5, Maktabi et al.2025; 6, Kim et al.2024; 7, Huang et al.2023; 8, Hsu et al.2023; 9, Monarchi et al.2025; 10, Oleru et al.2025; 11, Fang et al.2025; 12, Yang et al.2024; 13, Shi et al.2022; 14, Tighe et al.2022; 15, O’Neil et al. 2020

**Sensitivity and influence analyses**

**Figure S2a** Bivariate boxplot

**Figure S2b** Goodness-of-fit analysis

**Figure S2c** Bivariate normality assessment

**Figure S2d** Influence analysis

**Figure S2e** Outlier detection analysis
