# Supplementary material for: Diagnostic Accuracy of AI in Prediction and Assessment of Compromised Free Flaps: Systematic Review and Meta-Analysis
Source: J Med Internet Res. 2026 Sep 22;28:e91174. doi: 10.2196/91174 (PMC13596764; doi:10.2196/91174)
Supplement: Supplementary file 5 [file jmir-v28-e91174-s004.docx]

**FIGURE S3**

The following material accompanies the article **“Accuracy of Artificial Intelligence in Prediction and Detection of Compromised Free Flaps: A Systematic Review and Meta-Analysis”**

**Figure S3**

Deeks’ funnel plot asymmetry test evaluating potential publication bias among studies assessing AI–based diagnostic models for free flap compromise. Study labels correspond to the following studies: 1, Zhang et al. 2026; 2, He et al.2026; 3, Huang et al. 2026; 4, Kim et al. 2026; 5, Maktabi et al.2025; 6, Kim et al.2024; 7, Huang et al.2023; 8, Hsu et al.2023; 9, Monarchi et al.2025; 10, Oleru et al.2025; 11, Fang et al.2025; 12, Wang et al.2024; 13, Yang et al.2024; 14, Asaad et al.2023; 15, Shi et al.2022; 16, Tighe et al.2022; 17, O’Neil et al. 2020.

**Figure S3** Deeks’ funnel plot asymmetry test
